# Semi-analytical solution for bottomhole pressure transient analysis of a hydraulically fractured horizontal well in a fracture-cavity reservoir

**DOI:** 10.1038/s41598-022-26464-9

**Published:** 2022-12-21

**Authors:** Liu Hailong

**Affiliations:** 1 China University of Geosciences (Beijing), Haidian District, Beijing, 100083 China; 2 Northwest Center, Sinopec Petroleum Exploration and Development Research Institute, Haidian District, Beijing, 100083 China

**Keywords:** Environmental sciences, Hydrology

## Abstract

This paper study the role of hydraulic fracture properties on the transient bottomhole pressure (BHP) behavior of a horizontal well producing from a tight fracture-cavity reservoir. A combination of point source function, Laplace transformation and Perturbation transformation are used to obtain BHP step by step. Through literature comparison and numerical simulation, the results of BHP have a good consistency, which indicates the proposed method is scientific and reasonable. We divide the fluid flow into five stages, namely the wellbore storage stage, the karst cave fluid flows to the fracture stage, the radial flow stage of karst cave and fracture system, the matrix fluid flows to the fracture stage and the quasi-steady state stage. We come to the conclusion that the number of fractures and fracture direction mainly affect radial flow stage. In contrast, the length of horizontal subsection and skin factor mainly affect the karst cave fluid flows to the fracture stage. The matrix fluid flows to the fracture stage is more obvious when the fracture half-length and the horizontal segment spacing of the horizontal well are small. The study believes special attention should be paid to reforming the formations at both ends of the horizontal well. The advantage of this method is to incorporate well geometry (skin factor) and hydraulic fracturing design (fracture parameters), which is useful for well test interpretation through generating a new set of type curves. What’s more, this new method has the characteristics of easy calculation. The findings of this study can help for better understanding of well test analysis in fracture-cavity reservoir. However, the limitation of this study is that it is only suitable for this situation the horizontal well does not encounter karst caves and the karst caves in the reservoir are connected to the wellbore through fractures.

## Introduction

**Figure 1:**
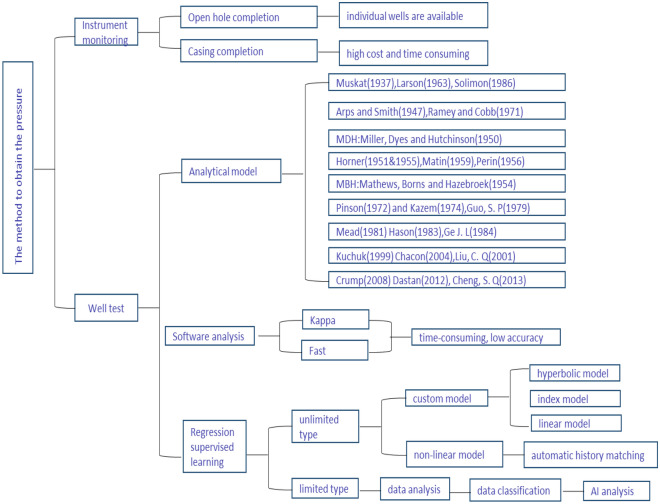
Summary of the methods used in well pressure measurement.

Bottom hole pressure (BHP), also known as flow pressure, is the pressure measured at the bottom of the well during production. BHP is a key index of producing wells. During different development stages, the production of oil wells has certain rules in different types of reservoirs and maximizing the development of oil well potential is the fundamental need of oilfield development. On the one hand, BHP is the most important parameter to determine the output of oil well. On the other hand, the oil flowing into the bottom hole is lifted to the surface by BHP, which is an important symbol of the self-injection capacity of the oil well. Therefore, transient analysis of BHP is very important for well production. Generally, there are two methods for obtaining BHP, one is direct measurement, and the other is indirect acquisition through well test, as shown in Fig. 1. This paper mainly focuses on the semi-analytical solution for BHP. The point source function is an effective tool to obtain BHP.

The idea of source functions originated from the heat conduction theory of the second half of the nineteenth century and was widely used by physicists in the 1930s. The method of point source is to use Green function method to solve indeterminate problem. Because the mathematical model of fluid seepage in porous media is similar to that of heat conduction in solid, many research results on heat conduction can be directly introduced into the seepage mechanics by analogy. In 1963, Warren and Root first introduced source function into the field of reservoir seepage^1^. In 1987, Brown et al. applied Laplace transform to establish point source function, line source function, and surface source function to solve the seepage problem of the dual-medium reservoir^2^. In 2000, Yang et al. discretized the fracture into small segments, and treated each segment as a slab source to deduce the slab-source function in tight oil reservoir^3^. In 2016, Zhang, et al. used Laplace transform and Fourier transform to establish a new surface function to present an analytical solution of horizontal well^4^.

Source function is one of the most important methods to solve the problem of reservoir seepage problem, and they are suitable to solve the reservoir seepage problems in vertical wells, horizontal wells, and fractured horizontal wells. In addition, they can provide an important theoretical basis for oil well productivity prediction and well test interpretation. However, with the development of carbonate fracture-cavity reservoirs, the traditional source function's application is greatly limited. Carbonate rock is a triple media reservoir with a certain stress-sensitive effect. Little research results have been reported at this point concerning the influence of stress sensitivity on the seepage of carbonate fracture-cavity in triple media.

As for BHP solution, many scholars have done a lot of research and made great progress. Literature survey found that although there are many ways to solve BHP, we can summarize them in three aspects:

**Single media pressure equation**: Based on Darcy's law, the relationship between productivity and pressure difference is established by considering linear or radial fluid flow ^5–10^. Generally, the relationship between the two is linear.

**Dual medium pressure equation**: Consider the fracture and matrix and the channeling flow between the fracture and matrix. The pressure equation is obtained according to the equal pressure and flow at the intersection of the fracture and the matrix interface^11–15^.

**Triple medium pressure equation**: Considering the fluid flow in the fracture, matrix, and karst cave, based on the positional relationship of the well, karst cave, and fracture, the pressure and flow rate at the intersection of the fracture, matrix, and karst cave is equal to obtain the pressure distribution^16–20^.

The analysis of fracture system is very important for identifying well geometry and reservoir structure. In the process of well test interpretation, there are many types of wellbore storage systems, and the corresponding interpretation models are also very complicated. The traditional diffusion equation is applied to describe the BHP behavior, however, evidence suggests that the pressure transient response of naturally fractured reservoirs typically exhibits strong heterogeneity. However, some recent research work has shown the homogeneity assumptions do not cover most situations^21^. On the one hand, fractal geometry is used to describe the heterogeneous reservoir^22,23^. On the other hand, Laplace transformation and Fourier cosine transformation are also effective to solve the diffusivity equation and analyze BHP behavior by considering the role of hydraulic fracture on BHP behavior^24,25^. With the development of numerical computation, many scholars also use numerical method to solve the BHP in complex heterogeneous reservoirs^26,27^. However, the diffusion process is historically related in a fractal reservoir, and the abnormal diffusion characteristics can’t be completely described through fractal models. In some of the literature, the fractal geometry and fractional calculus is introduced to describe fluid flow in fractal flow medium^28^.

Previous research on transient pressure analysis have done a lot of work, including different well types (horizontal and vertical well), different reservoir opening methods (partially penetrated and completely penetrated), and different boundary conditions (constant pressure boundary and closed boundary), which promote the development of well test analysis technology for different reservoirs. However, the development of a straight-forward solution for BHP behavior analysis in a fracture-cavity reservoir with a hydraulically fractured horizontal well, which can show all possible flow stages because of fracture heterogeneity, is still lacking in literature.

Besides, the literature survey found that the previous studies were mainly based on the classical Euclidean model, that is, most of them believed that fractures were uniformly distributed and that all fractures were the same, and the orientation of fractures is generally treated as simple, such as fractures parallel to each other or perpendicular to the horizontal section of the horizontal well. Fractures are of various orientations and sizes, and skin factor varies from fracture to fracture. Therefore, it is necessary to consider the actual situation of fracture and establish a pressure-solving model.

The point source function is an effective tool to solve the fracture heterogeneity. So far, the fracture network system no longer has the characteristics of embedded matrix and fractures at two different scales. This paper presents a new way to obtain the point source function and a new method of “three steps” to obtain BHP of a hydraulically fractured horizontal well under the constant pressure boundary, as shown below:

- **Step 1**: Through using the triple media model, the Pedrosa permeability calculation formula is applied to establish the seepage model of the triple media reservoir considering the formation stress sensitivity.
- **Step 2**: By perturbation transform and Laplace transform, the point source function considering stress sensitivity in fracture-cavity is obtained in Laplace space. The point source function in the infinite plate reservoir is obtained by the principle of mirror image and superposition.
- **Step 3**: The method of giving BHP under the constant pressure boundary is established through using the new point source function.

This paper proceeds as follows. First, Section “Model establishing” presents the physical model, and the assumptions and corresponding mathematical model is also present in section “Model establishing”. Then, section “Model solving” presents the process to solve the model including solution research, dimensionless transformation, equation solving, and discussion part. Section “Model Validation” validate the model in this paper available with the published analytical solution and numerical simulation, and the flow period is divided. Section “Sensitivity analysis” presents sensitivity research on the type curves. At last, section “Summary and Conclusions” presents the main conclusions.

## Model establishing

### Physical model

**Figure 2:**
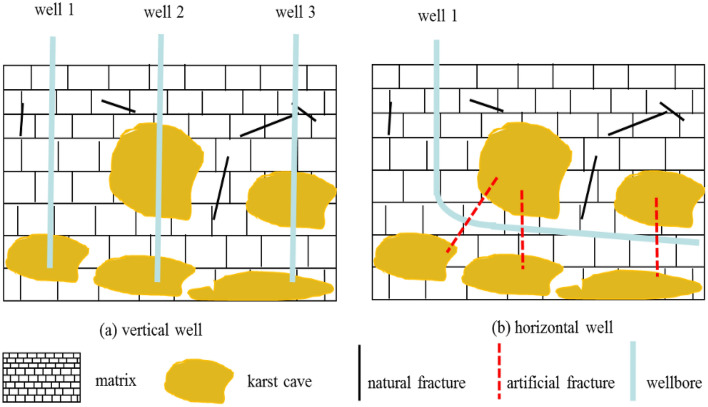
Schematic diagram of fracture-cavity reservoir.

The fracture-cavity reservoir develops fractures, and the distribution of fractures is complex, as shown in Fig. 2. The fracture-cavity reservoir has a triple medium property consisting of natural fractures, karst caves, and matrix. In Fig. 2a, at least three vertical wells are required to develop the reserves of five karst caves. In Fig. 2 (b), by hydraulic fracturing, only one horizontal well can develop the reserves of these five caves. Compared with vertical well, horizontal wells have lower cost and control larger drainage area in the exploitation of fracture-cavity reservoir.

In Fig. 2b, the well is drilled into the matrix of the reservoir, hydraulic fracturing must be used to build the artificial fractures, which can establish the communication channel between karst caves and wellbore. This paper aims to solve the dynamic response of BHP under the condition that the horizontal section of the well is located in the matrix of the tight fracture-cavity reservoir.

For the Shunbei Oilfield, the fracture-cavity body of the reservoir is relatively developed. Its bedrock porosity is minor, and the permeability is low. It is a somewhat typical tight limestone with a very high carbon–oxygen ratio. The fluid flow in the reservoir mainly depends on the communication between fractures and karst caves^29^. Due to the random distribution of fractures and karst caves, it is generally impossible to form a good fracture- cavity system, so the heterogeneity of fracture-cavity reservoirs is particularly strong^30^. The Shunbei Oilfield is a particular fracture-cavity reservoir and has some unique features^31^. On one hand, many unexposed karst fracture-cavity reservoirs are developed along the fault zone, on the other hand, the reservoir storage space, storage type, fluid properties and distribution all show diversity and complexity. The horizontal heterogeneity is substantial, but the vertical connectivity is good, and the matrix has no storage and permeability capabilities. Due to the development of faults in the Shunbei oilfield, it is common to set wells in the reservoir matrix and communicate karst caves and natural fractures by hydraulic fracturing in the drilling design.

**Figure 3:**
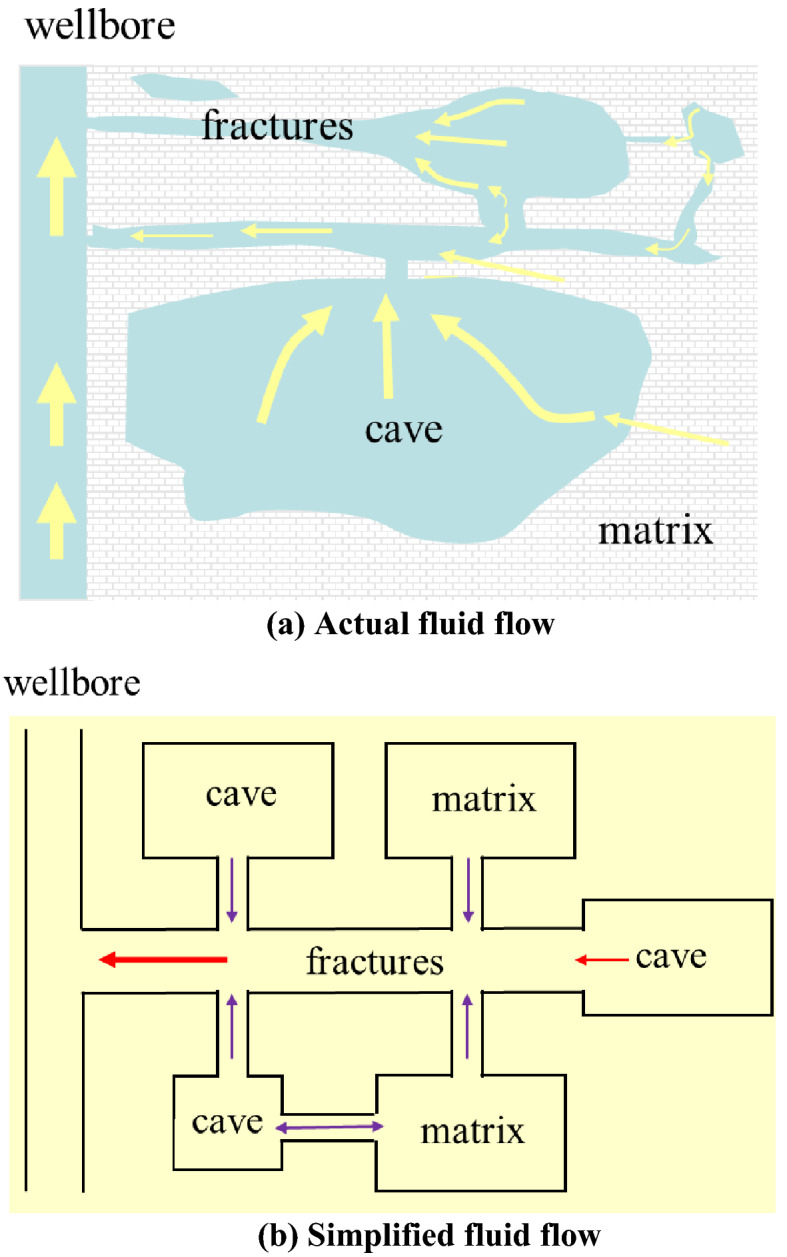
Simplified diagram of fluid flow.

Fractured-cavity reservoirs have diverse reservoir spaces and complex fluid flow. The actual fluid flow diagram in the reservoir is shown in Fig. 3a. To simplify calculations and facilitate mathematical modeling, the basic flow of the reservoir is simplified as the flow shown in Fig. 3b. Meanwhile, the following assumptions are made:

1. The oil reservoir is composed of the karst cave system, matrix system, and fracture system. The karst cave system is the main storage space, and the fracture system is the main flow channel. All the well production comes from the influx of the fracture system;
2. Considering the permeability sensitivity of natural fracture system, it is assumed that the permeability of matrix system is constant;
3. There is a point source in the oil reservoir. The initial pressure is equal everywhere in the oil reservoir, and the reservoir temperature is constant during the production process of the oil well.
4. The reservoir fluid is slightly compressible, and the influence of gravity and capillary force is not considered. The porosity of the reservoir and fluid viscosity is constant.

**Figure 4:**
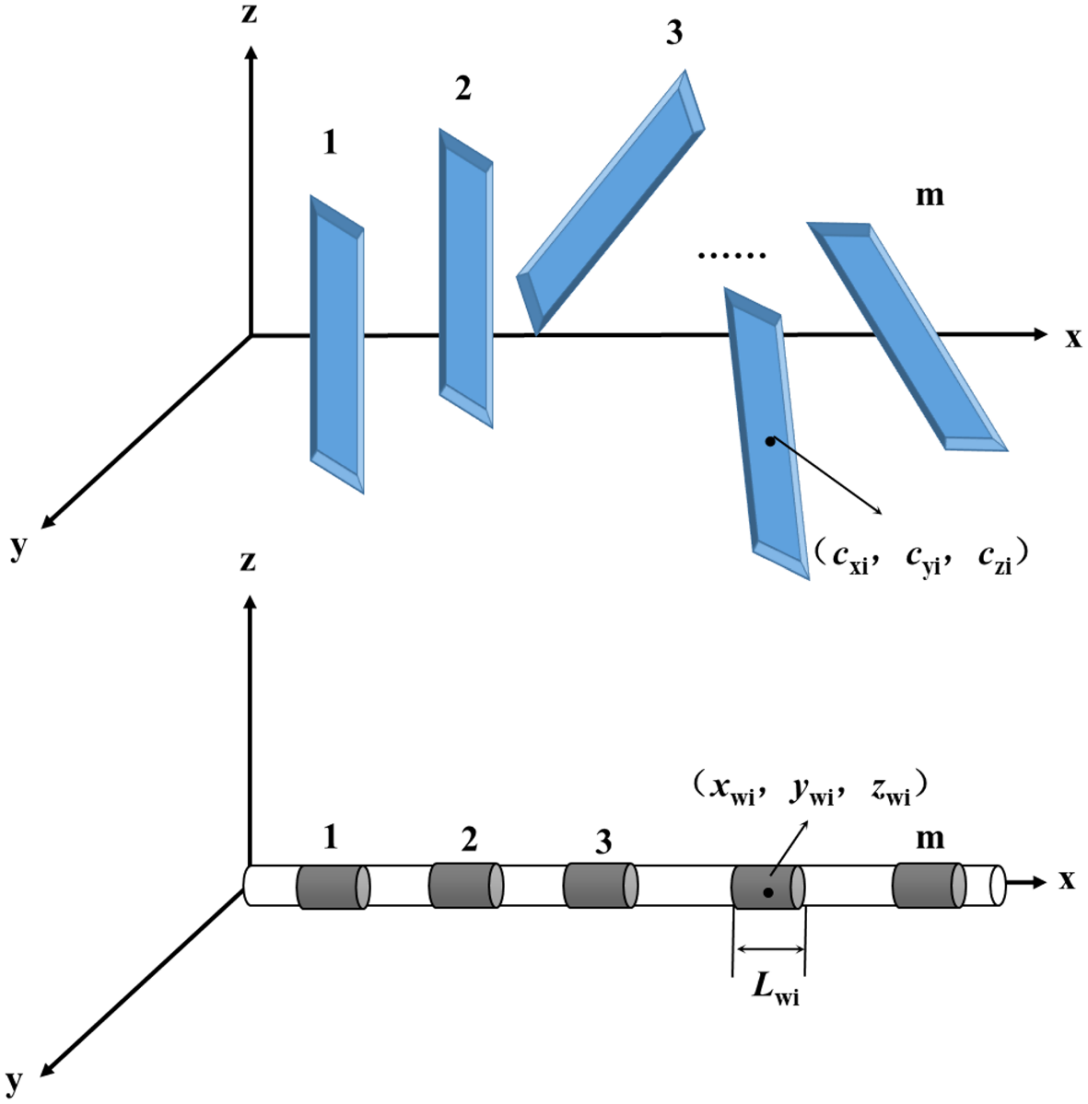
Schematic diagram of fracture distribution and horizontal section division of horizontal wells.

Since the orientation of fractures is not uniform, the number of fractures in the reservoir is assumed to be m, and the horizontal section of the horizontal well is divided into m sections accordingly. The center of each horizontal well segment is parallel to the fracture center along the z-axis. After division, the schematic diagram is shown in Fig. 4. Without considering the channeling between fractures, each fracture can be regarded as the source phase of the corresponding horizontal well segment. Then, considering the fluid flow of a fracture and the related horizontal section, the pressure model under a single fracture can be established. Finally, the pressure distribution of the entire horizontal well can be obtained according to the pressure superposition.

### Mathematical model

Based on the above assumptions, the center of a single fracture is located at (x_0_, y_0_, z_0_), and the governing equation of the fracture is shown as follows^18^:

1 $$ \begin{gathered} \frac{{k_{x} }}{\mu }\frac{{\partial^{2} p_{f} }}{{\partial x^{2} }} + \frac{{k_{y} }}{\mu }\frac{{\partial^{2} p_{f} }}{{\partial y^{2} }} + \frac{{k_{z} }}{\mu }\frac{{\partial^{2} p_{f} }}{{\partial z^{2} }} + \frac{{8qw_{x} w_{y} w_{z} }}{\mu }f(x,y,z) \hfill \\ = (\phi c)_{f} \frac{{\partial p_{f} }}{\partial t} + (\phi c)_{m} \frac{{\partial p_{m} }}{\partial t} + (\phi c)_{v} \frac{{\partial p_{v} }}{\partial t} \hfill \\ \end{gathered} $$

*f(x,y,z)* is the source (sink) term, and its expression is shown as below.

2 $$ f(x,y,z) = \frac{1}{{8w_{x} w_{y} w_{z} }}\iiint\limits_{v} {\cos \alpha \cos \beta \cos \chi  \cdot \delta }\left[ {\left( {x - x_{0} } \right)\left( {y - y_{0} } \right)\left( {z - z_{0} } \right)} \right]dv  $$

3 $$ (x_{0} - w_{x} )(y_{0} - w_{y} )(z_{0} - w_{z} ) \le v \le (x_{0} + w_{x} )(y_{0} + w_{y} )(z_{0} + w_{z} ) $$

If $\Delta p = p_{i} - p$, then the change trend of $\Delta p$ and $p$ is consistent. The pressure term $p$ in the governing Eq. (1) can be directly replaced with pressure difference term $\Delta p$.

4 $$ \begin{gathered} \frac{{k_{x} }}{\mu }\frac{{\partial^{2} \Delta p_{f} }}{{\partial x^{2} }} + \frac{{k_{y} }}{\mu }\frac{{\partial^{2} \Delta p_{f} }}{{\partial y^{2} }} + \frac{{k_{z} }}{\mu }\frac{{\partial^{2} \Delta p_{f} }}{{\partial z^{2} }} + \frac{{8qw_{x} w_{y} w_{z} }}{\mu }f(x,y,z) \hfill \\ = (\phi c)_{f} \frac{{\partial \Delta p_{f} }}{\partial t} + (\phi c)_{m} \frac{{\partial \Delta p_{m} }}{\partial t} + (\phi c)_{v} \frac{{\partial \Delta p_{v} }}{\partial t} \hfill \\ \end{gathered} $$

Similarly, the governing equation of the matrix system can be obtained as the Eq. (5)

5 $$ (\phi c)_{m} \frac{{\partial \Delta p_{m} }}{\partial t} = \sigma_{m} \frac{{k_{m} }}{\mu }(\Delta p_{f} - \Delta p_{m} ) $$

The governing equation of the karst cave system is obtained as the Eq. (6)

6 $$ (\phi c)_{v} \frac{{\partial \Delta p_{v} }}{\partial t} = \sigma_{v} \frac{{k_{v} }}{\mu }(\Delta p_{f} - \Delta p_{v} ) $$

When considering the stress sensitivity of the natural fracture system, the natural fracture permeability decreases with the decrease of formation pressure. The natural fracture permeability can be expressed as:

7 $$ k_{x} = k_{xi} e^{{ - \alpha (p_{i} - p)}} = k_{xi} e^{ - \alpha \Delta p} $$

## Model solving

### Solution research

Literature survey found that there are four main methods to solve the Eq. (4). First: assuming that the reservoir pressure reaches the bottom of the well instantaneously, based on the Green's function, the analytical solution of the pressure in the Laplace space domain is obtained^5,8,11^; Second: a numerical solution to the pressure in the real domain is obtained based on the separation of variables and the numerical difference discrete method^3,12,13^; Third: Assume that the bottom hole flow reaches a pseudo-steady state, ignore the pressure and time changes, only consider the relationship between pressure and space, and use the successive steady-state method to obtain the pressure distribution^2,9^; Fourth: get the pressure in the Laplace space through dimensionless change, Laplace transform, and Fourier transform and then obtain the real domain pressure numerical solution through numerical inversion^14,17,18^. This paper considers the triple-media and stress-sensitive characteristics of fracture-cavity reservoirs, applying perturbation transformation, Laplace transforms, mirror image principle and Poisson summation formula, a new point source function for fracture-cavity reservoirs is established. Simultaneously, considering the problem that the conventional source function cannot calculate the formation stress sensitivity, the numerical solution of the BHP of the horizontal well is solved.

### Equation solving

To transform a practical physical problem into a pure mathematical one, we must first transform dimensional quantities (physical quantities) into dimensionless quantity (mathematical quantity), so that all analysis, calculation is applicable to all the scale of measurement. This process is called dimensionless processing. The dimensionless treatment of this paper is shown in Appendix 1. Through dimensionless transformation of Eq. (4), we can get:

8 $$ e^{{ - \Delta p_{f} }} \frac{1}{{r_{D}^{2} }}\frac{\partial }{{\partial r_{D} }}\left( {r_{D}^{2} \frac{{\partial \Delta p_{fD} }}{{\partial r_{D} }}} \right) + \frac{4\pi }{{N^{2} }}f(r_{D} ) = \omega_{f} \frac{{\partial \Delta p_{fD} }}{{\partial t_{D} }} + \omega_{m} \frac{{\partial \Delta p_{mD} }}{{\partial t_{D} }} + \omega_{v} \frac{{\partial \Delta p_{vD} }}{{\partial t_{D} }} $$

Laplace transformation and Perturbation transformation are used to solve Eq. (8) and obtain the point source function for the problem under study. The detail of the derivation is shown in Appendix 2.

The pressure distribution calculation formula of instantaneous point source function per unit strength is obtained as follow:

9 $$ \overline{{\omega_{0} }} = \left[ {\frac{{4\pi \ln sf(r_{D} )}}{{N^{2} }} - sf(s)} \right] + \frac{{\overline{q} }}{{[(\phi c_{t} )_{f} + (\phi c_{t} )_{m} + (\phi c_{t} )_{v} ]}}\frac{{\exp \left( { - r_{D} \sqrt {sf(s)} } \right)}}{{4\pi L^{3} r_{D} }} $$

Please note that the point source is located at the origin of coordinates.

### Discussion

#### Point source function is not at the origin of coordinates

If the point source function is not at the origin of coordinates, but at (x_wD_, y_wD_, z_wD_) , first define the following expression:

10 $$ R_{D} = \sqrt {(x_{D} - x_{wD} )^{2} + (y_{D} - y_{wD} )^{2} + (z_{D} - z_{wD} )^{2} } $$

Then the calculation formula of pressure distribution generated by the unit source function is as follows:

11 $$ \overline{{\psi_{0} }} = \left[ {\frac{{4\pi \ln sf(R_{D} )}}{{N^{2} }} - sf(s)} \right] + \frac{{\overline{q} }}{{[(\phi c_{t} )_{f} + (\phi c_{t} )_{m} + (\phi c_{t} )_{v} ]}}\frac{{\exp ( - R_{D} \sqrt {sf(s)} )}}{{4\pi L^{3} R_{D} }} $$

If:

12 $$ \overline{S} = \frac{{\exp ( - R_{D} \sqrt {sf(s)} )}}{{4\pi L^{3} R_{D} }} $$

Then, the time–space solution of the second part in Eq. (11) is:

13 $$ \psi_{0} { = }\frac{{\widetilde{q}}}{{[(\phi c_{t} )_{f} + (\phi c_{t} )_{m} + (\phi c_{t} )_{v} ]}}S(t_{D} ) $$

By applying the superposition principle, the solution of continuous point source function can be obtained as follows:

14 $$ \psi_{0} { = }\frac{{\widetilde{q}}}{{[(\phi c_{t} )_{f} + (\phi c_{t} )_{m} + (\phi c_{t} )_{v} ]}}\int\limits_{0}^{t} {\widetilde{q}} (\tau )S(t_{D} - \tau )d\tau = \frac{{\mu L^{2} }}{{k_{if} }}\int\limits_{0}^{{t_{D} }} {\widetilde{q}} (\tau_{D} )S(t_{D} - \tau_{D} )d\tau_{D} $$

Based on the property of convolution, Laplace transform of Eq. (13), and we can get:

15 $$ \overline{{\psi_{0} }} = \frac{{\mu \overline{{\widetilde{q}}} }}{{4\pi k_{if} L}}\frac{{\exp \left( { - R_{D} \sqrt {sf(s)} } \right)}}{{R_{D} }} $$

So, the Eq. (11) can be rewritten as:

16 $$ \overline{{\psi_{0} }} = [\frac{{4\pi \ln sf(R_{D} )}}{{N^{2} }} - sf(s)] + \frac{{\mu \overline{{\mathop q\limits^{\sim } }} }}{{4\pi k_{if} L}}\frac{{\exp ( - R_{D} \sqrt {sf(s)} )}}{{R_{D} }} $$

#### Upper and lower closed boundary

**Figure 5:**
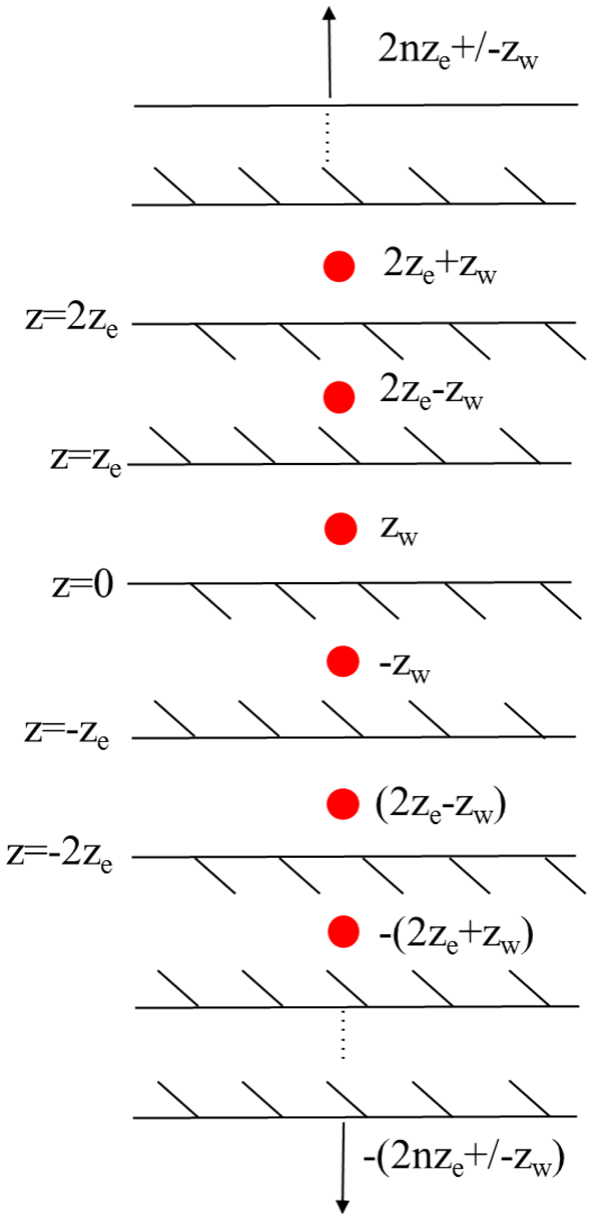
Schematic diagram of mirroring principle of closed boundary reservoir.

The pressure of the fracture-cavity reservoir is solved based on the mirror image principle. It is assumed that the point source is in a plate fracture-cavity reservoir with closed upper and lower boundaries. The reservoir height is $h$ . The position of the point source is $z_{w}$. Schematic diagram of closed boundary mirroring principle, as shown in Fig. 5.

After mirror inversion, the position of the point source is:

17 $$ \left\{ \begin{gathered} 2nh_{D} - z_{Dw} \hfill \\ 2nh_{D} + z_{Dw} \hfill \\ \end{gathered} \right.{\kern 1pt} {\kern 1pt} {\kern 1pt} {\kern 1pt} {\kern 1pt} {\kern 1pt} {\kern 1pt} {\kern 1pt} {\kern 1pt} {\kern 1pt} {\kern 1pt} (n = 0, \pm 1, \pm 2, \pm 3,..., \pm \infty ) $$

Based on the superposition principle, the point source function of upper and lower closed plate fracture-cavity reservoir can be expressed as:

18 $$ \begin{gathered} \overline{{\psi_{0} }} = \frac{{\mu \overline{{\widetilde{q}}} }}{{4\pi k_{f} L}}\sum\limits_{n = - \infty }^{ + \infty } {\left\{ {\frac{{\exp ( - \sqrt {sf(s)} \sqrt {(x_{D} - x_{wD} )^{2} + (y_{D} - y_{wD} )^{2} + (z_{D} + z_{wD} - 2nh_{D} )^{2} } )}}{{\sqrt {(x_{D} - x_{wD} )^{2} + (y_{D} - y_{wD} )^{2} + (z_{D} + z_{wD} - 2nh_{D} )^{2} } }}} \right.} \hfill \\ {\kern 1pt} {\kern 1pt} {\kern 1pt} {\kern 1pt} {\kern 1pt} {\kern 1pt} {\kern 1pt} {\kern 1pt} {\kern 1pt} {\kern 1pt} {\kern 1pt} {\kern 1pt} {\kern 1pt} {\kern 1pt} {\kern 1pt} + \left. {\frac{{\exp ( - \sqrt {sf(s)} \sqrt {(x_{D} - x_{wD} )^{2} + (y_{D} - y_{wD} )^{2} + (z_{D} - z_{wD} - 2nh_{D} )^{2} } )}}{{\sqrt {(x_{D} - x_{wD} )^{2} + (y_{D} - y_{wD} )^{2} + (z_{D} - z_{wD} - 2nh_{D} )^{2} } }}} \right\} \hfill \\ \end{gathered} $$

The Possion relation, trigonometric function and difference product formula can be used to transform the above equation, and the following equation can be obtained:

19 $$ \overline{{\psi_{0} }} = \frac{{\mu \overline{{\widetilde{q}}} }}{{2\pi k_{f} Lh_{D} }}\left[ {K_{0} (r_{D} \sqrt {sf(s)} ) + 2\sum\limits_{n = 1}^{ + \infty } {K_{0} \left( {r_{D} \sqrt {sf(s) + \left( {\frac{n\pi }{{h_{D} }}} \right)^{2} } } \right)} \cos n\pi \frac{{z_{D} }}{{h_{D} }}\cos n\pi \frac{{z_{wD} }}{{h_{D} }}} \right] $$

#### Upper and lower constant pressure boundaries

Similarly, when the upper and lower boundaries have constant pressure, the solution of the point source function is:

20 $$ \overline{{\psi_{0} }} = \frac{{\mu \overline{{\widetilde{q}}} }}{{\pi k_{f} Lh_{D} }}\left[\sum\limits_{n = 1}^{ + \infty } {K_{0}  \left(r_{D} \sqrt {sf(s) +\left(\frac{n\pi }{{h_{D} }}\right)^{2} } \right)} \sin n\pi \frac{{z_{D} }}{{h_{D} }}\sin n\pi \frac{{z_{wD} }}{{h_{D} }}\right] $$

When z = 0 is the closed boundary, and z = h is the constant pressure boundary, the solution of the point source function is:

21 $$ \overline{{\psi_{0} }} = \frac{{\mu \overline{{\widetilde{q}}} }}{{\pi k_{f} Lh_{D} }}\left[ {\sum\limits_{n = 1}^{ + \infty } {K_{0} \left( {r_{D} \sqrt {sf(s) + \left( {\frac{(2n - 1)\pi }{{2h_{D} }}} \right)^{2} } } \right)} \cos (2n - 1)\frac{\pi }{2}\frac{{z_{D} }}{{h_{D} }}\cos (2n - 1)\frac{\pi }{2}\frac{{z_{wD} }}{{h_{D} }}} \right] $$

The boundary types of fracture-cavity reservoirs are diverse, so we will not discuss them one by one here.

#### Solution of obtaining BHP

Based on the above three types of point source functions with different upper and lower boundary properties, seepage solution equations can be constructed under different conditions. For the physical model in this article, the length of the horizontal section of the horizontal well is L, and the BHP solving steps are as follows:

① Firstly, it is assumed that the length and height of a certain fracture are 2*L*_fi_ and *h*_wi_. Then, according to the boundary type of fracture-cavity reservoir (the above pressure boundary is taken as an example to illustrate), Formula (20) integrates x in the interval of (x_w_-L_f_, x_w_ + L_f_) , and then integrates z in the interval of(z_w_-h_w_/2, z_w_ + h_w_/2) . The expression of the pressure of a single fracture in the horizontal section of the horizontal well can be obtained.
② According to the superposition principle, the pressure generated by each fracture in the corresponding horizontal section is superimposed to obtain the Laplace space solution of the total pressure.
③ According to the solution of Laplace space, the Stehfest method is used to carry out numerical inversion, and the numerical solution of real space is obtained.

## Model validation

### Literature comparison and validation

**Figure 6:**
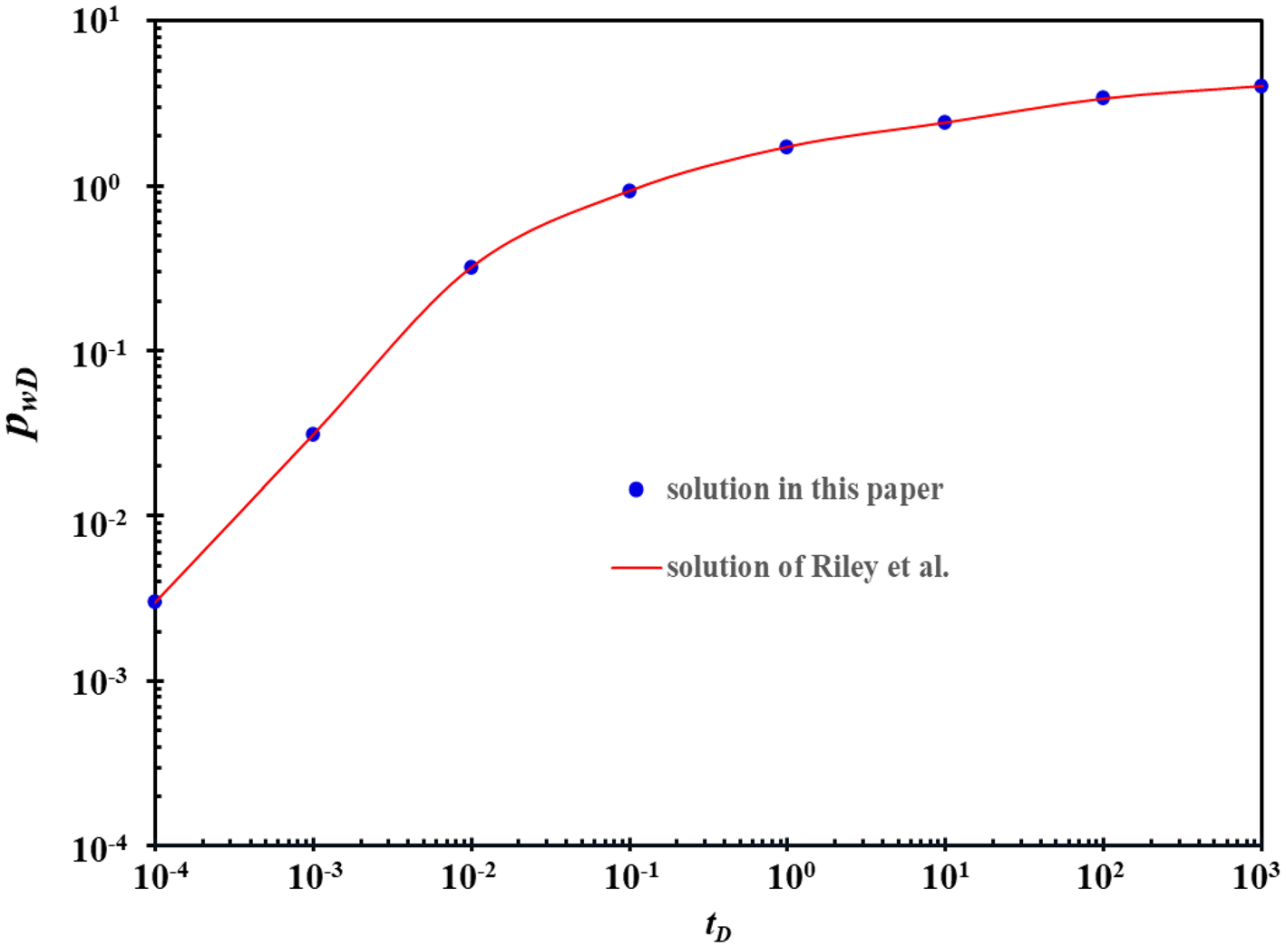
Comparison of calculation results.

Riley et al. solved the analytical solution of BHP in horizontal wells with only one fracture supply^20^. For the convenience of comparison, it is assumed that the fracture in the physical model of horizontal well in this paper is one. The angle between the fracture and the horizontal section is 90°. The fracture locates in the center of the horizontal wellbore. To be consistent with the conditions in the literature, the influence of karst caves is not considered. Other basic reservoir parameters are shown in Table 1. The calculation results of Riley et al. and those in this paper are shown in Fig. 6. Figure 6 shows that the results of the two have a good consistency. Riley et al. is a special case of the model in this paper, so the model in this paper is correct.

**Table 1:** Basic reservoir parameters.

Parameter	Value	Parameter	Value
Original saturation pressure (MPa)	44	Effective thickness (m)	30
Formation oil viscosity (mPa s)	0.45	Horizontal length	400
Original reservoir pressure (MPa)	65	Crude oil density (g/cm^3^)	0.729
Number of fractures	1	Comprehensive compression coefficient (10^–4^1/MPa)	13.8

### The numerical simulation validation

For comparison purposes, the number of fractures is assumed to be 5. The relevant parameters of fractures are shown in Table 2. The reservoir is a plate reservoir with upper and lower boundary with constant pressure. By simulating the productivity of horizontal wells with different fracture numbers and fracture locations, the BHP of the corresponding horizontal wells (the simulation solution) was obtained and compared with the pressure obtained from numerical inversion in this paper (this paper solution).

**Table 2:** List of fracture parameters.

Parameter	Fracture 1	Fracture 2	Fracture 3	Fracture 4	Fracture 5
Location	(500,500)	(550,500)	(600,500)	(650,500)	(700,500)
Angle with horizontal segment	30°	45°	60°	75°	90°
Fracture width	10 mm	8 mm	6 mm	4 mm	2 mm
Fracture half-length	200 m	160 m	120 m	80 m	40 m
Fracture height	5 m	5 m	5 m	5 m	5 m
Initial horizontal permeability	100 mD	100 mD	100 mD	100 mD	100 mD
Initial vertical permeability	500 mD	500 mD	500 mD	500 mD	500 mD

**Figure 7:**
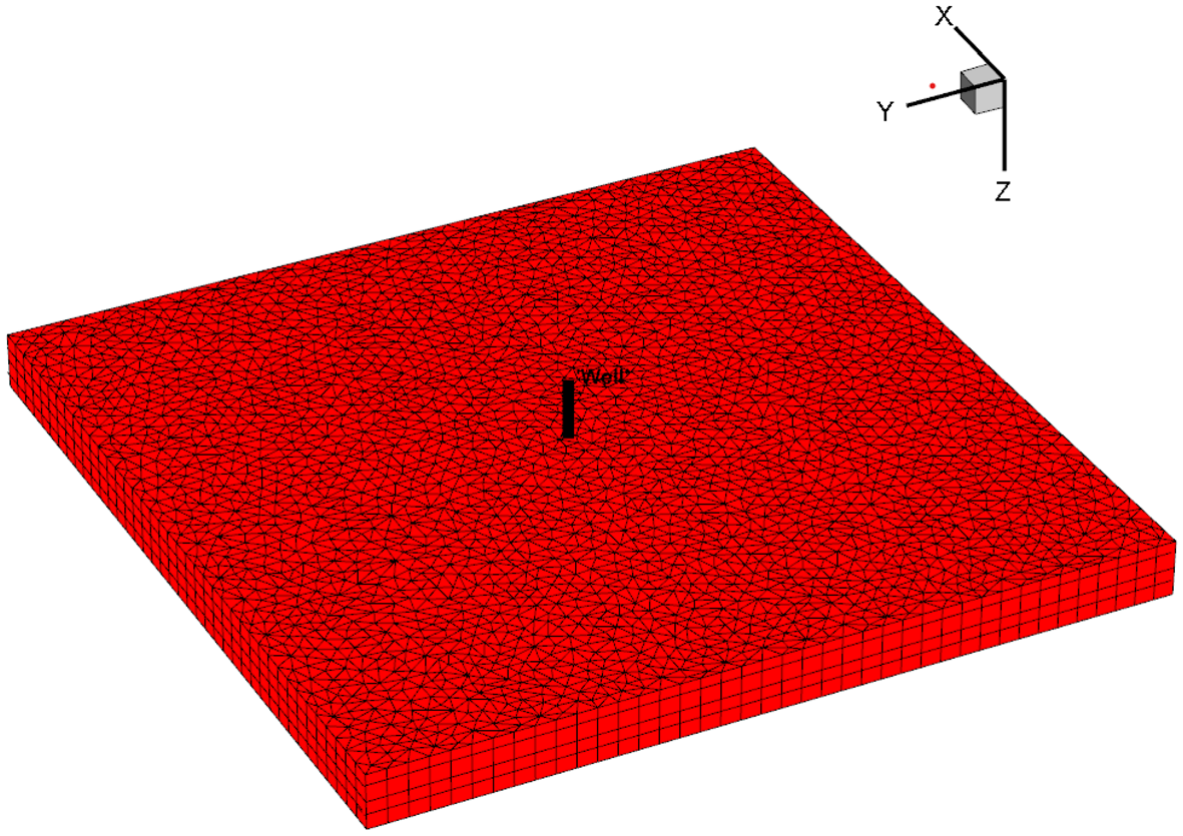
Schematic diagram of well location and grid division.

The E300 module in Eclipse 2017 is designed for triple-media fracture-cavity reservoirs. To meet the assumptions of the model derived in this paper, the numerical model is set as follows: The width and length of the heterogeneous fracture-cavity reservoir are 1000 m, and there is a horizontal well in the center of the reservoir with a horizontal section length of 200 m, as shown in Fig. 7. The triangulation mesh method is adopted to divide meshing and ensure that each fracture has at least three meshing to describe the heterogeneity of formation fluid. If the fracture is short, local mesh encryption should be performed at the fracture. The plane mesh step size is 20 m, and the vertical mesh step size is 5 m. Then the total number of grids is 50*50*16 = 40,000. Other parameters required for numerical simulation are shown in Table 3. The numerical simulation and calculation results in this paper are shown in Table 4. Table 4 shows that the relative error is controlled within 5% under the basic error, which is consistent with the allowable error range^19^, indicating that the method we provide is reliable.

**Table 3:** Basic reservoir parameters.

Parameter	Value	Parameter	Value
Original saturation pressure (MPa)	44	Formation water density (g/cm^3^)	1.0
Formation oil viscosity (mPa s)	0.45	Formation oil volume coefficient	1.612
Permeability modulus (1/MPa)	0.0012	Crude oil density (g/cm^3^)	0.729
Surface oil viscosity (mPa s)	1.71	Oil–water interface depth (m)	3100
Formation water viscosity (mPa s)	0.50	Formation water volume coefficient	1.102
Coefficients of compression of rock (10^–4^1/MPa)	5.0	Formation oil compression coefficient (10^–4^1/MPa)	34
Horizontal well length(m)	200	Coefficients of compression of formation water (10^–4^1/MPa)	4.2
Formation thickness(m)	80	Fracture storage capacity ratio	0.15
Initial formation pressure(Mpa)	65	Karst cave storage volume ratio	0.25
Number of fractures	5	Matrix storage capacity ratio	0.60
Radial permeability of fracture(mD)	100	Radial permeability of matrix(mD)	1

**Table 4:** Comparison of calculation results.

Production time (d)	Productivity (m^3^/d)	The simulation solution (MPa)	This paper solution (MPa)	Relative error (%)
30	33.137992	51.807017	50.148556	3.201228513
60	32.651338	50.204722	48.549205	3.297532451
90	32.164684	48.963757	47.317118	3.362975190
120	31.67803	47.962682	46.336766	3.389960553
150	30.91002	47.130427	45.522290	3.412099364
180	30.89413	46.292744	44.474019	3.928747451
210	30.68332	45.876605	43.841381	4.436300376
240	29.35811	44.004106	42.051935	4.436338282
270	27.86354	43.377195	41.211481	4.992749070
300	25.92476	42.3976848	41.850898	1.289662873
330	22.11243	41.4181746	40.698511	1.737554605
360	19.15212	40.4386644	39.546125	2.207144111

### Flow characteristics analysis

**Figure 8:**
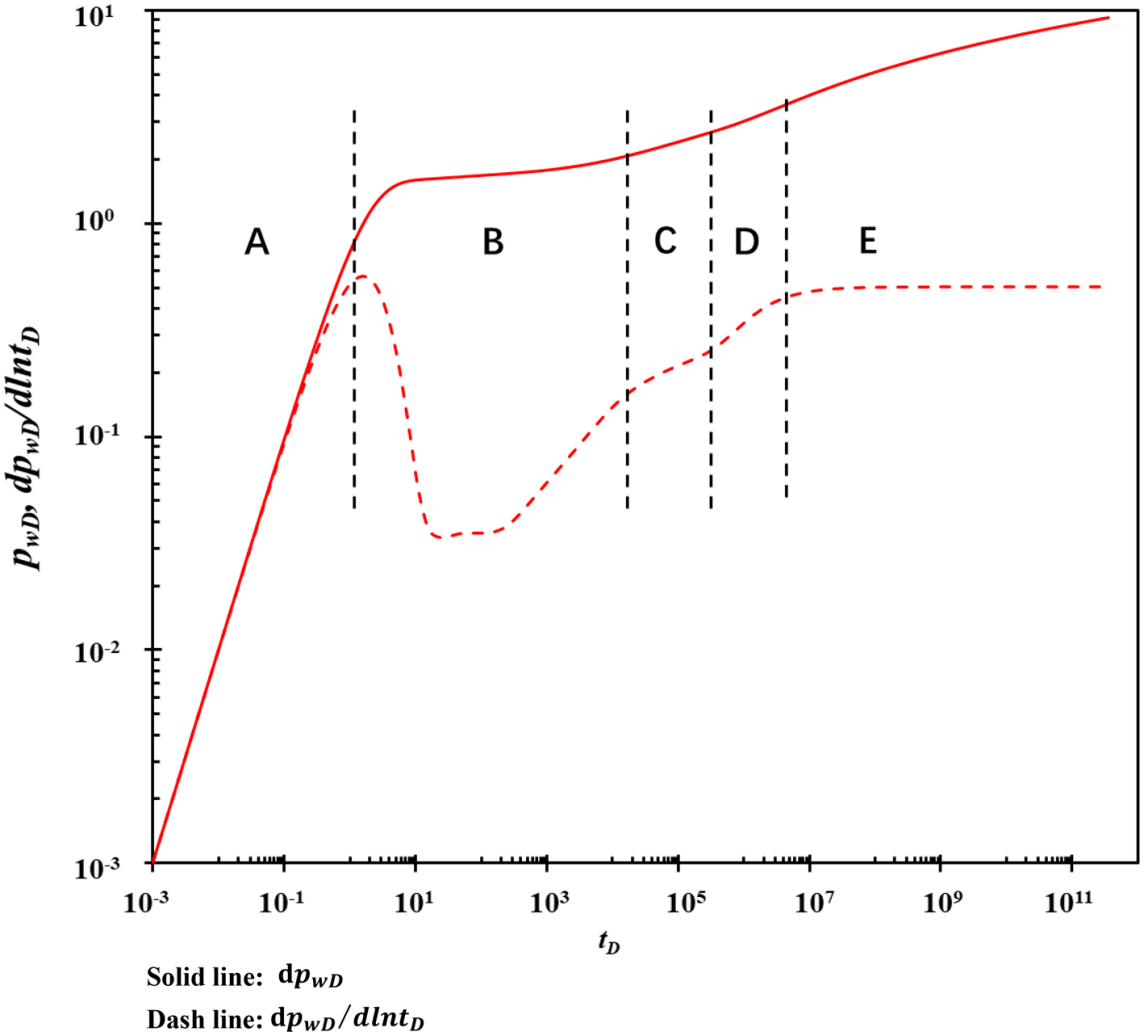
The flow division schematic of the type curves.

A fracture-cavity reservoir with a rectangular outer boundary has a horizontal well in the center. The number of fractures is 4. The length of the horizontal section of the horizontal well is 400 m. *k*_x_ = *k*_y_, *k*_z_/*k*_x_ = 100. The wellbore storage factor is 0.001, and the skin factor is 1. Based on the basic parameters in Table 1, the dimensionless pressure and pressure derivative were calculated, as shown in Fig. 8. Figure 8 shows that the fluid flow process of the triple media fracture-cavity reservoir can be divided into five stages, considering fracture orientation and stress sensitivity.

- **Stage A: The wellbore storage stage**. The pressure and pressure derivative curves coincide, and the asymptotic analysis shows that the slope of the curve is about 1. This stage is mainly affected by the wellbore storage effect.
- **Stage B: The karst cave fluid flows to the fracture stage**. Generally, the permeability of the karst cave system is greater than that of the fracture system. As the fracture system supplies fluid to the wellbore, the pressure of the fracture system drops. Then the karst cave system first supplies fluid to the fracture to supplement the pressure of the fracture system. A “dent” appears in the curve.
- **Stage C: Radial flow stage of karst cave and fracture system**. The slope of the pressure derivative curve is about 0.5 when the fracture fluid supplied by the karst cave system is in equilibrium with the wellbore fluid supplied by the fracture system. This stage is short or infrequent due to the presence of a matrix, which also supplies fracture fluid.
- **Stage D: Matrix fluid flows to the fracture stage**. When the fluid in the karst cave and fracture system is extracted, the pressure of the two systems decreases continuously until it is less than the pressure of the matrix system, and the fluid in the matrix begins to flow to the fracture and karst cave under the action of pressure difference.
- **Stage E: Quasi-steady state stage**. When the flow between matrix—fracture, karst cave—fracture, and fracture—wellbore is in equilibrium, the system reaches the quasi-steady state. The pressure derivative curve approximates a horizontal line, and the asymptotic analysis shows that the horizontal line is about 0.5.

## Sensitivity analysis

According to the method and process of solving BHP in this paper, the sensitivity analysis of the influencing factors of pressure and pressure derivative is carried out based on the principle of the single factor variable. The influences of fracture number, fracture angle, fracture half-length, skin factor, and horizontal well segment length, and horizontal well segment spacing on pressure and pressure derivative are analyzed in detail.

### Horizontal segment spacing of horizontal well

**Figure 9:**
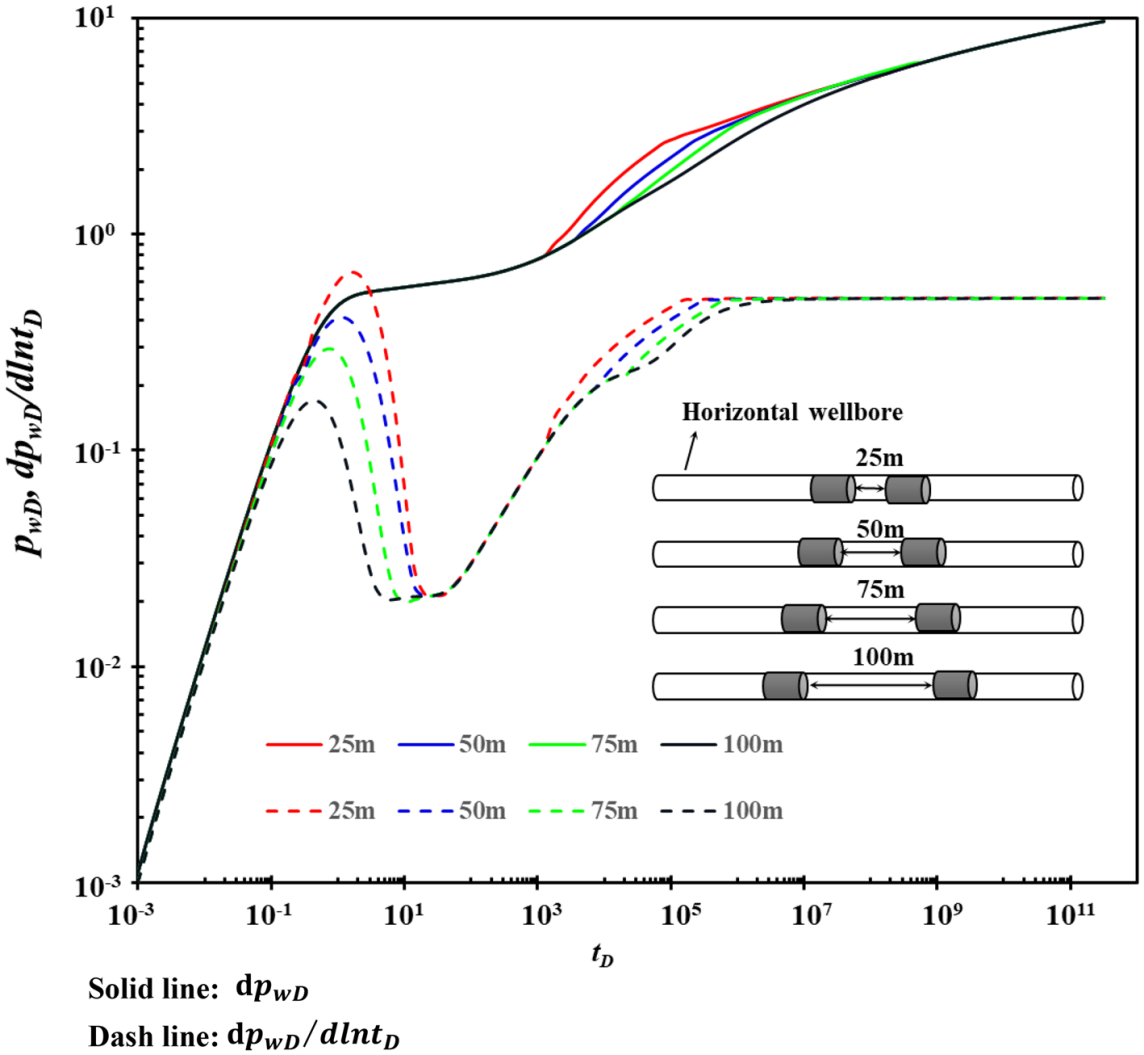
The effect of segment spacing on type curves.

When the total length of the reservoir shot by the horizontal well is fixed, the entire drainage area communicating with the fractures outside the wellbore is certain. The influence of horizontal segment spacing on the type curves is shown in Fig. 9. Figure 9 shows that horizontal segment spacing mainly affects stages B and C. The larger the interval between the horizontal segments of the horizontal well, the longer it takes for fractures to supply the wellbore. The more difficult the channeling flow between the karst cave and fracture will be.

When the horizontal segment spacing is large, a new “platform” appears on the pressure derivative curve in stage C, with a horizontal line of about 0.25. The asymptotic analysis shows that the new “platform” height depends on the number of segments in the horizontal well. In general, when the number of segments in the horizontal well is n, the corresponding horizontal line of the new “platform” is about 1/n.

Infield well testing, it isn't easy to have stage E if the test time is not long enough. Therefore, during well test interpretation, the actual permeability can be obtained by multiplying the permeability k explained in stage C by the number of segments of horizontal wells.

### Horizontal well segment length

**Figure 10:**
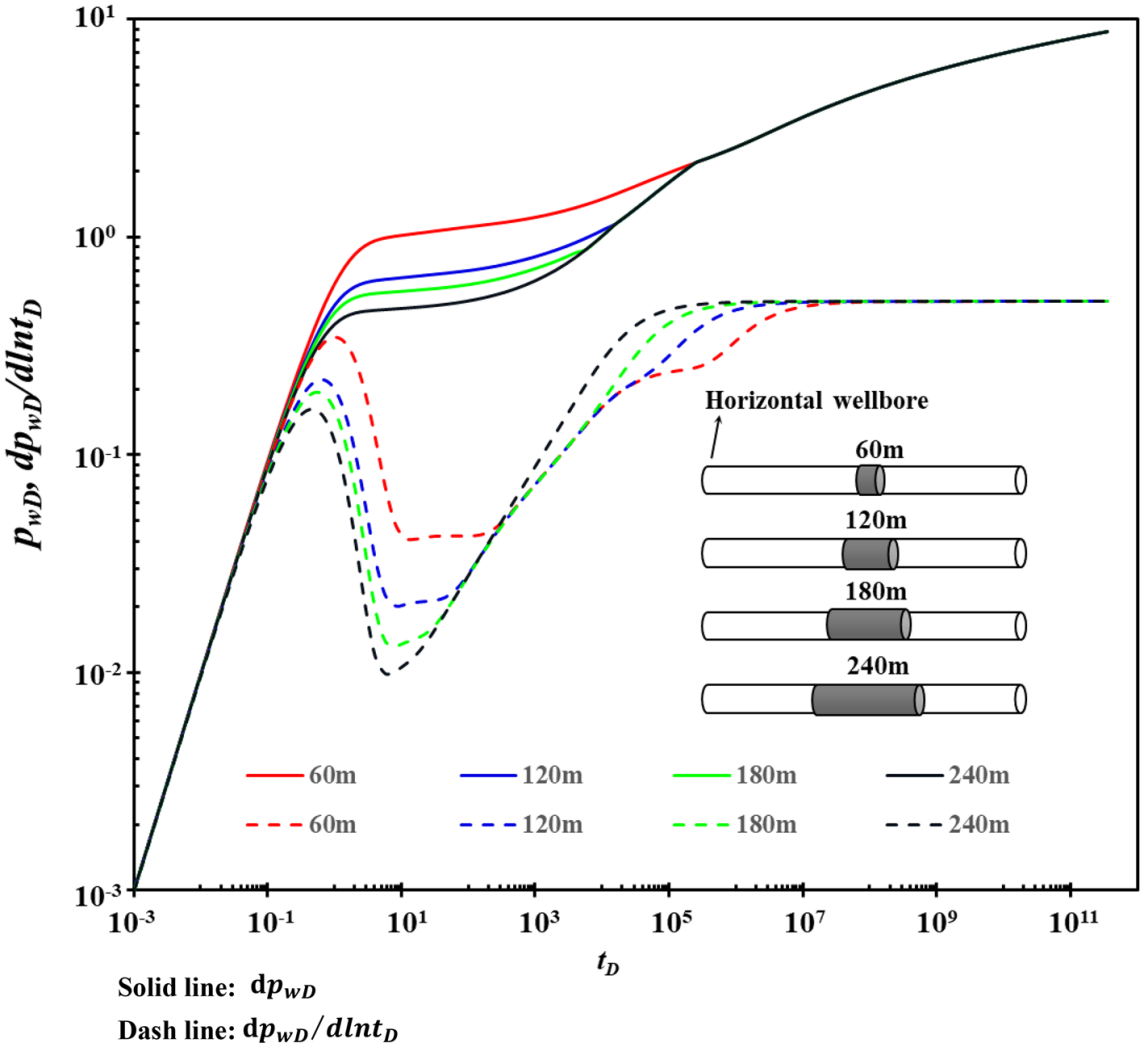
The effect of section length on type curves.

The influence of different horizontal segment lengths on the type curves is shown in Fig. 10. Figure 10 shows that segment length mainly affects stage B. The longer the segment length is, the lower the “hump” is, and the earlier stage B is. However, the shorter the duration of stage B is, and the lower the value of the pressure derivative is and more quickly recovered to 0.5. The larger the reservoir interval opened by horizontal wells, the more favorable the flow of reservoir fluid to the wellbore, and the earlier the well reservoir effect will disappear.

**Figure 11:**
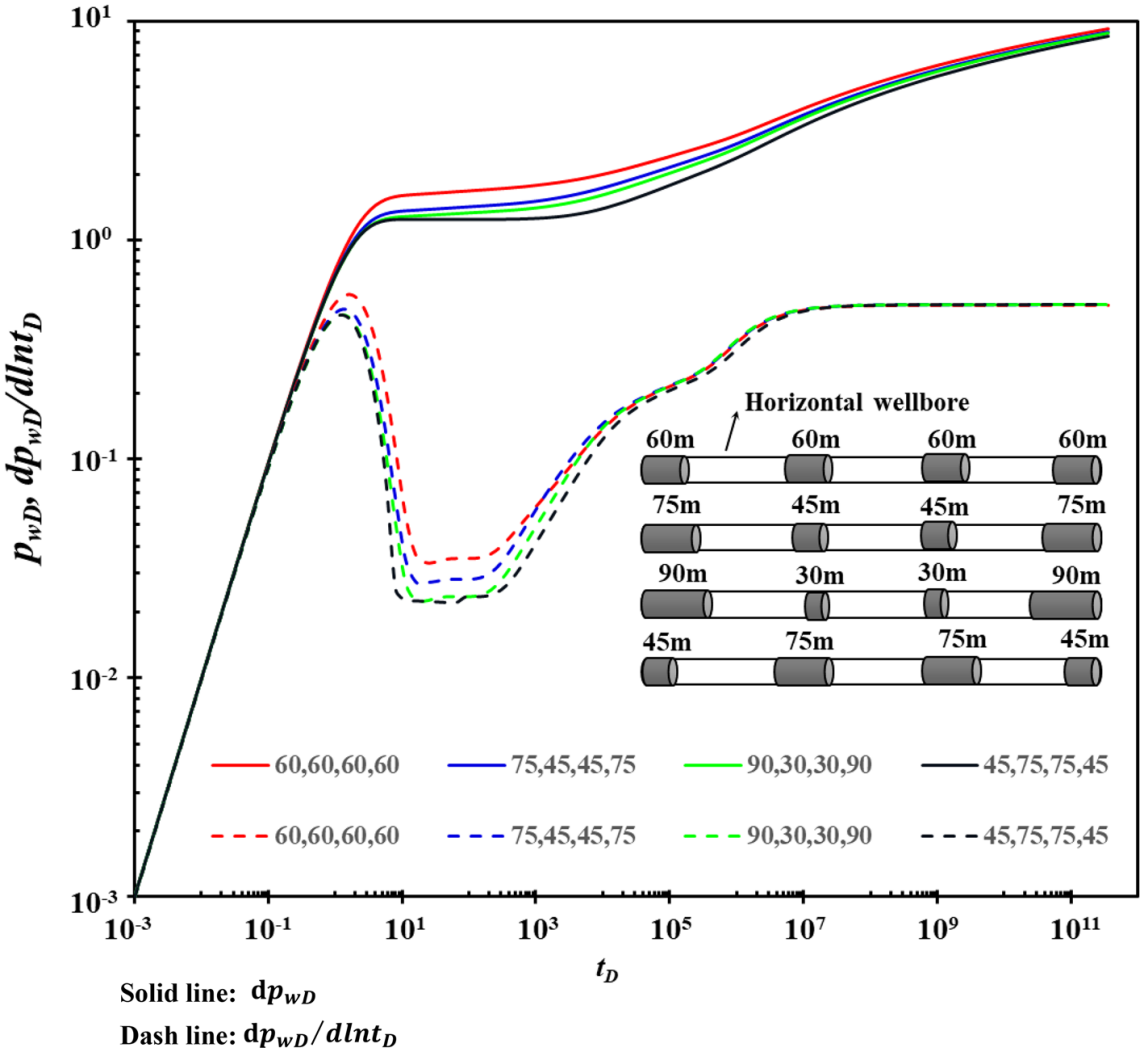
The effect of section length and distribution mode on type curve.

When the total length of the horizontal segment is fixed, the influence of each segment's length and distribution mode on the type curves is shown in Fig. 11. Figure 11 shows that when the section length at both ends of the horizontal well increases, the pressure drop generated becomes smaller, conducive to horizontal well production. Therefore, in the actual perforation process, it is suggested to increase the production section at the toe and foot ends of the horizontal well to increase the supply of fractures to the horizontal wellbore and improve the horizontal wellbore productivity.

### Skin factor

**Figure 12:**
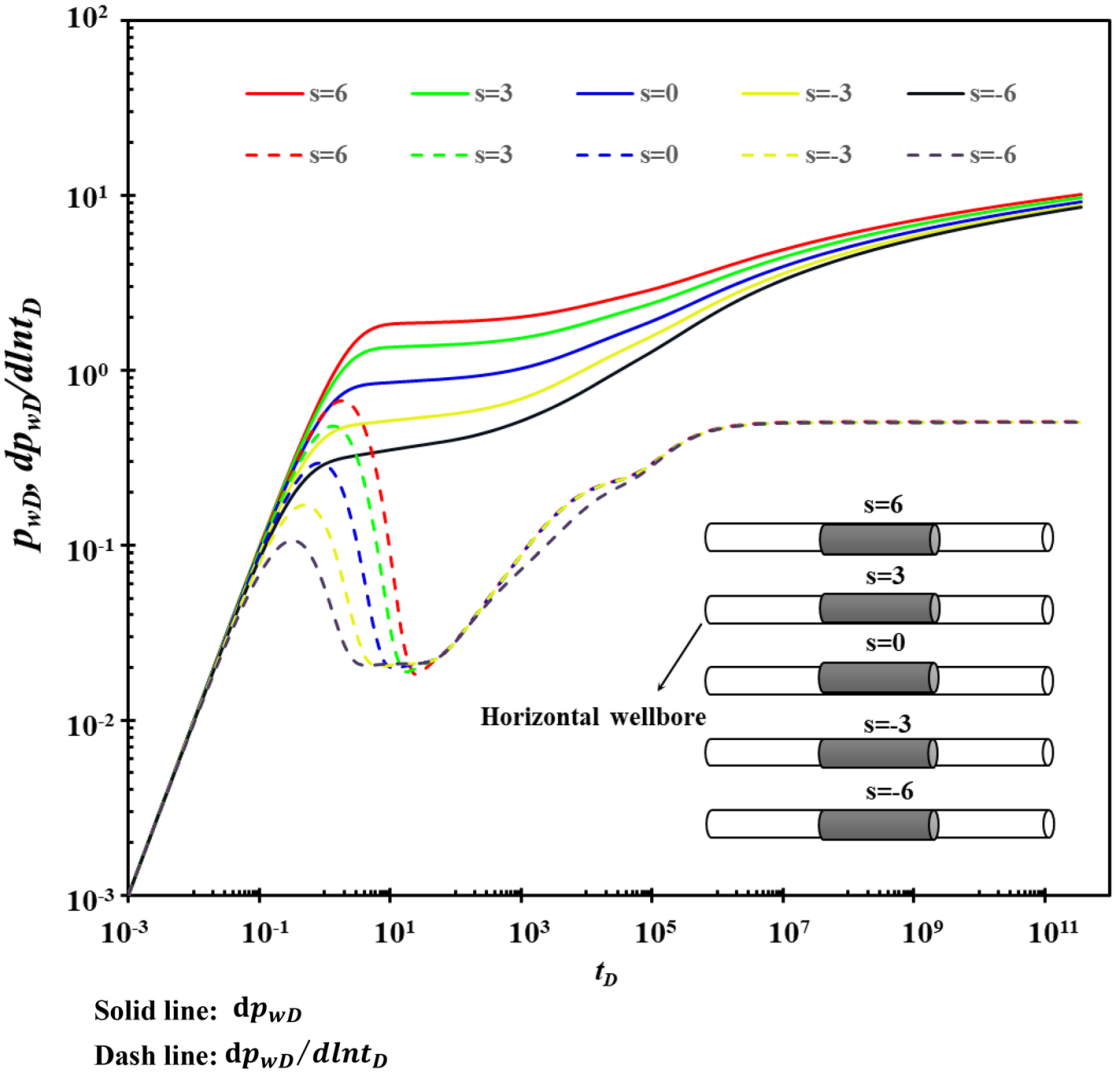
The effect of skin factor on type curves.

The influence of the skin factor on the type curves is shown in Fig. 12. Figure 12 shows that the larger the skin factor is, the more serious the wellbore pollution is. What's more, the longer the duration of stage A is, and the later the appearance of stage B is, and the higher the “hump” is. Stage C is covered in varying degrees, and the skin factor affects the radial flow of the fracture.

**Figure 13:**
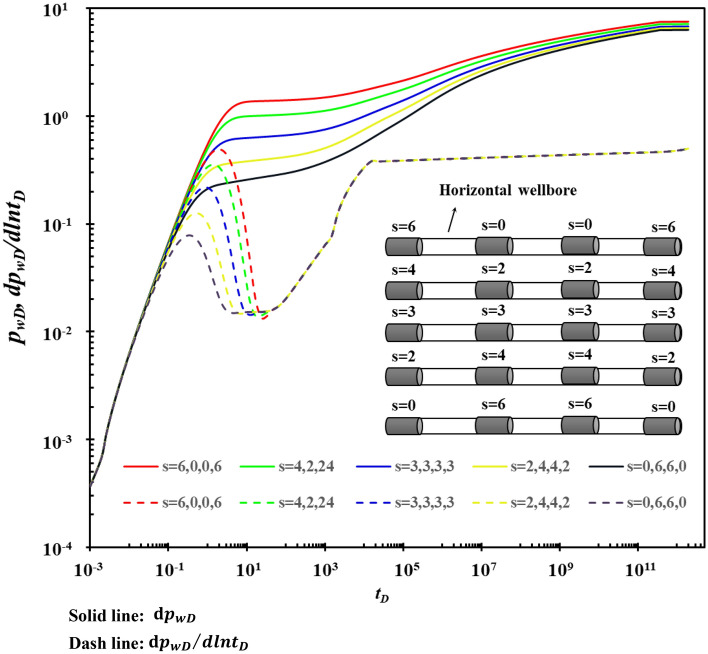
The effect of the size and distribution of the skin coefficient on type curves.

When the sum of the total skin factor of each segment is constant, the influence of the size and distribution of the skin factor of each segment on the type curves is shown in Fig. 13. Figure 13 shows that the skin factor causes different flow rates in each section, and the non-uniformly distributed skin factor produces different pressure responses at the bottom of the well.

When the pollution at both ends of the horizontal well is relatively serious, the pressure drop loss caused is greater, which is not conducive to improving the production of the horizontal well. Therefore, when using horizontal wells to implement reservoir stimulation measures, special attention should be paid to modifying the formations at both ends of the horizontal well to eliminate pollution near the wellbore.

### Fracture number

**Figure 14:**
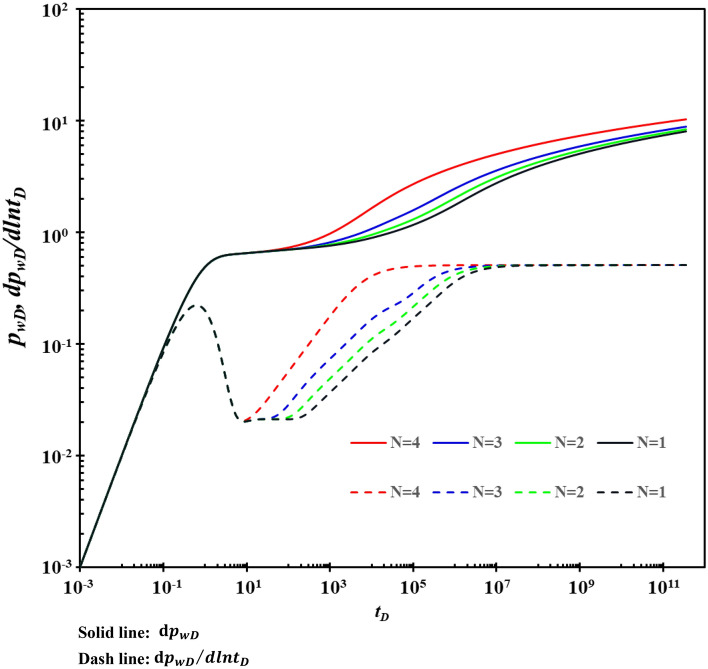
The effect of the number of fractures on type curves.

The influence of the fracture number on the type curves is shown in Fig. 14. Figure 14 shows that the fracture number mainly affects stage C. When there are many fractures, and the fractured-vuggy reservoir has a fracture network, stage D will be covered by stage C. To a certain extent, the matrix does not directly supply the karst caves. Instead, the matrix directly supplies the fractures, mainly caused by the development of a fracture network in the reservoir. The more developed the fracture network, the seepage characteristics of the triple-medium fracture-cavity reservoir are more similar to the dual-media. The matrix/cavities in the reservoir directly supply the fractures and then flow to the wellbore. This flow mode is more conducive to pressure transmission and production increase.

### Fracture direction

**Figure 15:**
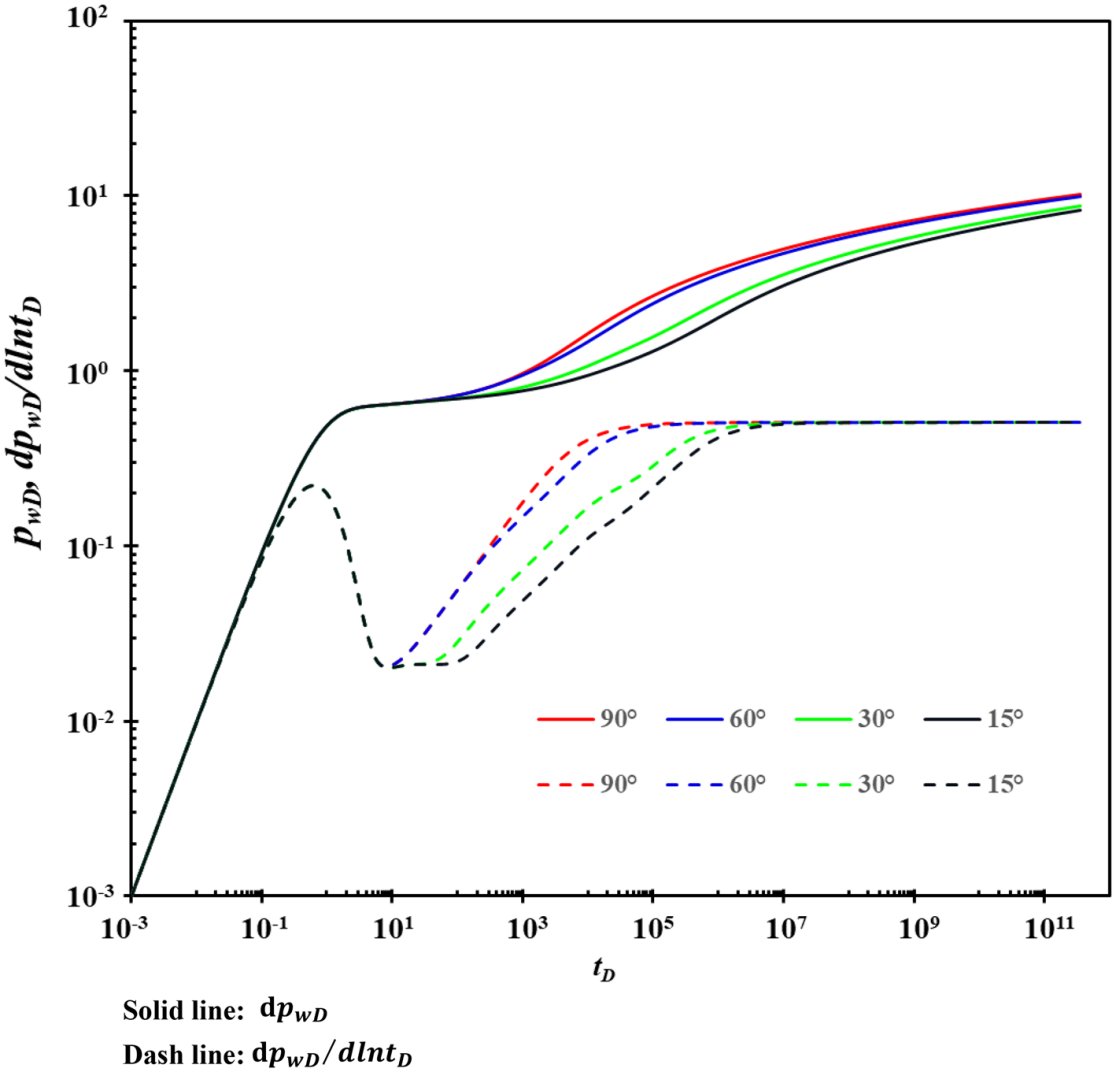
The effect of the fracture direction on type curves.

When the angle between the fracture and the horizontal wellbore changes, different fracture angles have different effects on the type curves, as shown in Fig. 15. Figure 15 shows that the fracture direction mainly affects stage C. When the fracture is perpendicular to the wellbore, the pressure drop is smaller, and the pressure transmission is faster, which is more conducive to improving the productivity of horizontal wells. Because the drainage area is larger, and more fluid in the reservoir flows through the fractures to the wellbore at the same time. The perforating gun should be parallel to the horizontal wellbore during fracturing as much as possible, and then fractures perpendicular to the wellbore should be generated.

### Fracture half-length

**Figure 16:**
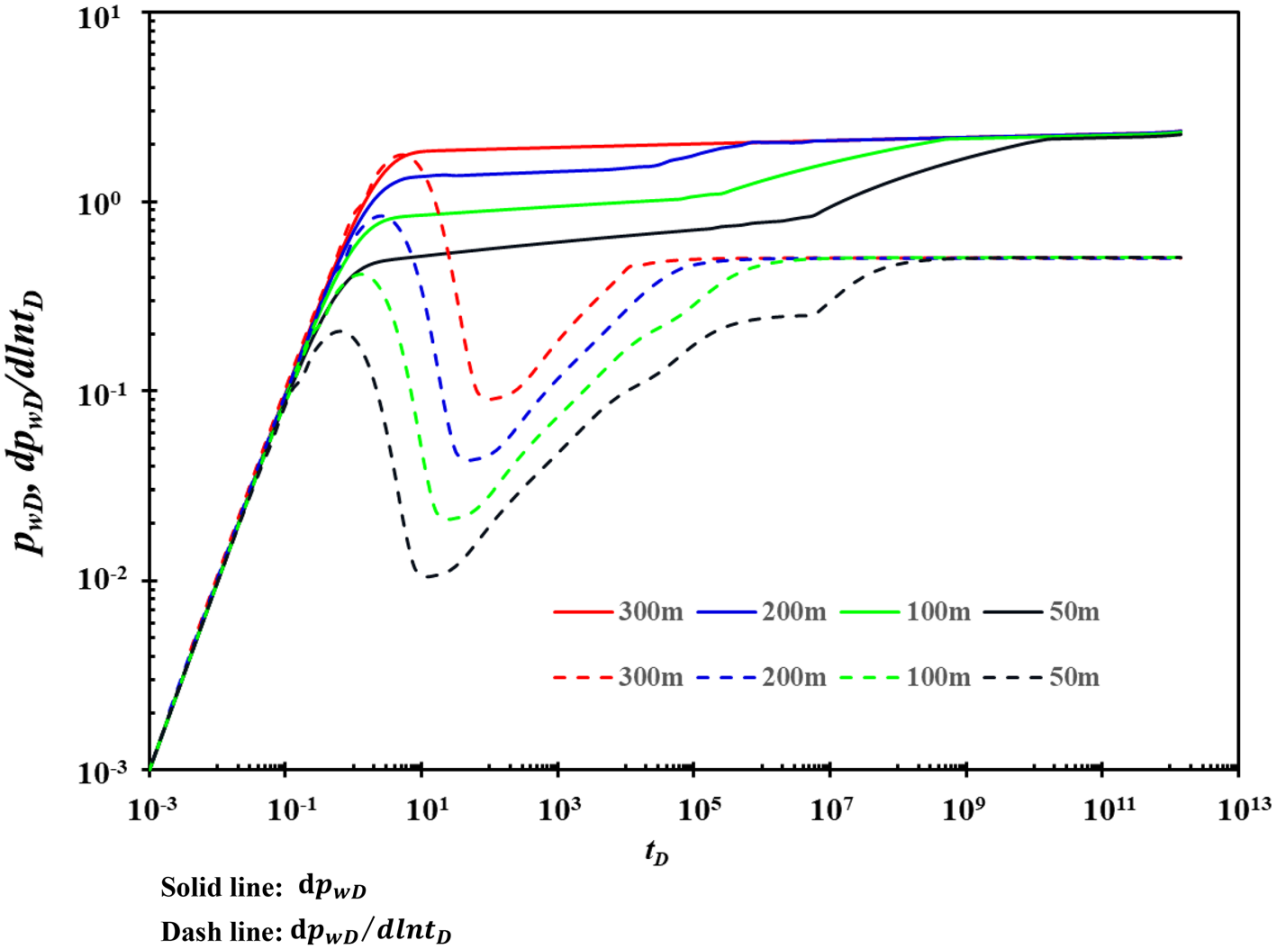
The effect of fracture half-length on type curves.

The effect of the fracture half-length on the type curves is shown in Fig. 16. Figure 16 shows that the fracture half-length mainly affects stage B and stage C. When the fracture half-length is longer, the pressure drop is smaller, which is most conducive to production. When the fracture half-length is very small (≤ 50 m), stage D is more obvious because the fracture half-length is small, the fluid in the matrix cannot flow directly to the fracture. It must flow to the karst cave first and then flows to the fracture through the karst cave. The drainage area in the reservoir is limited, and the pressure spreads more slowly. Therefore, in the design of hydraulic fracturing, the fracture half-length should be greater than 50 m as far as possible.

### Permeability modulus

**Figure 17:**
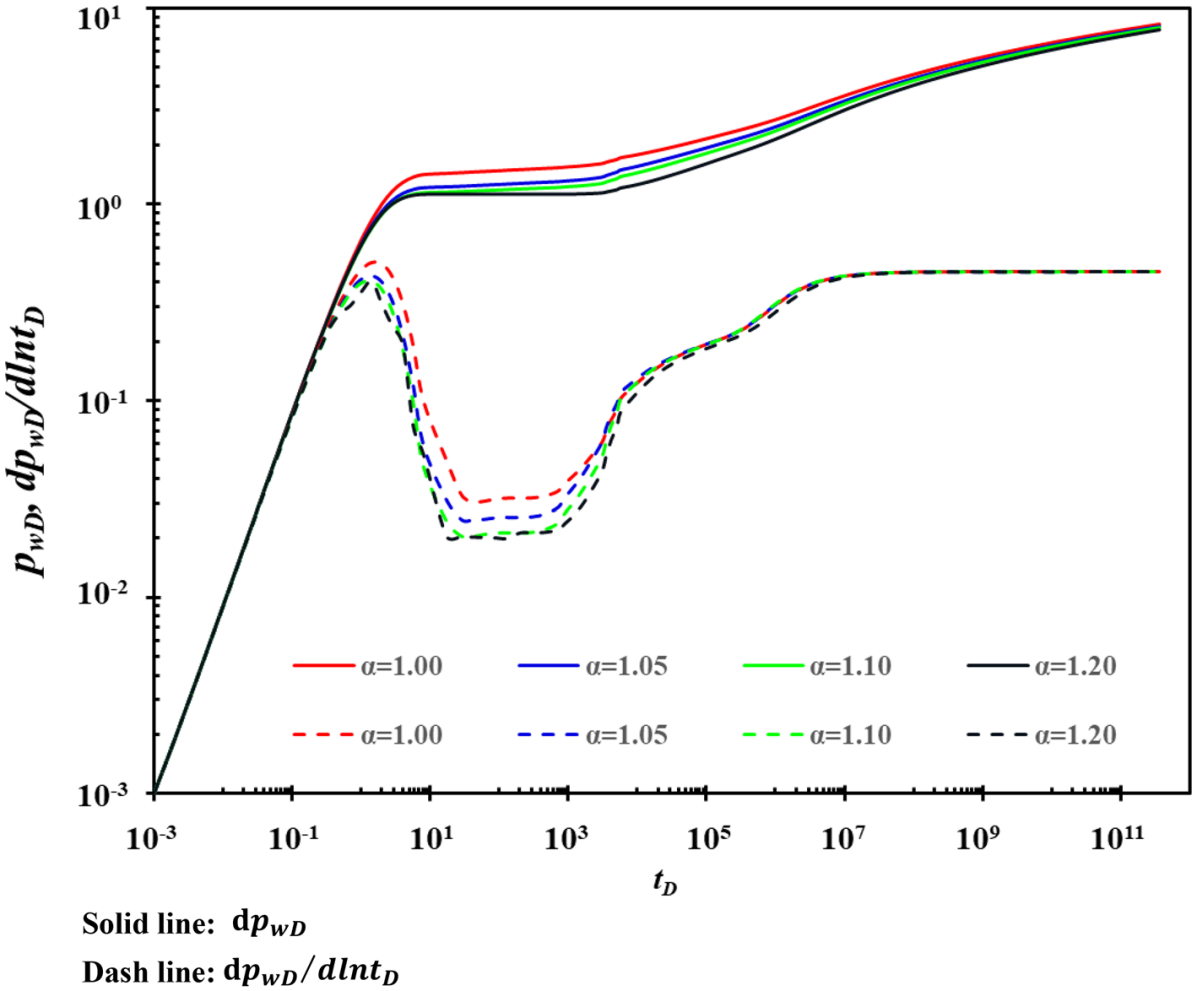
The effect of permeability modulus on type curves.

The effect of the permeability modulus (α) on the type curves is shown in Fig. 17. Figure 17 shows that the permeability modulus mainly affects stage B and stage C. The larger the permeability modulus is, the greater the minimum value of the curve in stage B is. The permeability modulus also affects the maximum value of stage C. The smaller the permeability modulus is, the greater the maximum value of the curve in stage C is. Permeability modulus has similar properties to rock compressibility. Since the compressibility is of very small order of magnitude (generally in the order of 10^–4^), it can be regarded as a constant in engineering applications. However, the order of magnitude of permeability modulus is much larger than that of compression coefficient, and its variation should be considered according to the actual situation in application, which is not simply regarded as a constant in fracture-cavity reservoirs.

### Well test interpretation

Through conventional linear analysis of well test, wellbore storage coefficient can be obtained from the curve of early pure wellbore storage stage, and formation coefficient, formation effective permeability, skin coefficient and reservoir pressure can be obtained from the curve of late radial flow stage. Some initial values obtained by conventional well test interpretation are substituted into the well test model, and the initial parameters are constantly modified through using the genetic simulated annealing algorithm, until the theoretical curve completely fits the measured curve. The whole well test interpretation process is a semi-automatic analysis process. For the triple media reservoir model with fracture and wellbore connection, the parameters to be explained in well test are mainly wellbore storage coefficient, skin coefficient, fracture permeability, channeling-flow coefficient between matrix and fracture, channeling-flow coefficient between karst cave and fracture, elastic storage ratio of matrix and elastic storage ratio of fracture.

**Figure 18:**
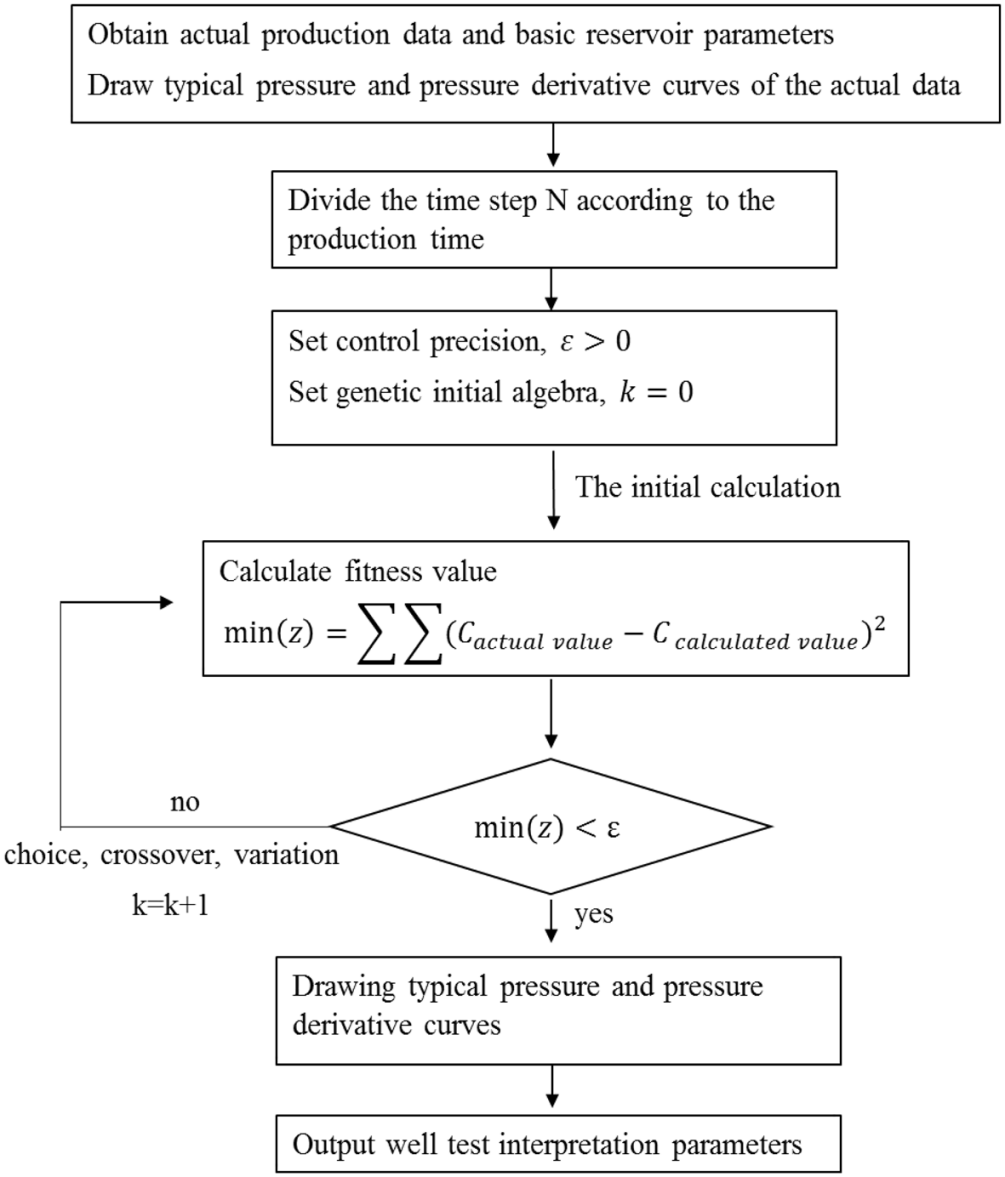
Curve fitting flow chart.

Genetic Algorithm (GA) is a computational model of biological evolution that simulates the natural selection and Genetic mechanism of Darwin's biological evolution^32^. It is a method to search for the optimal solution by simulating the natural evolution process. Its essence is an efficient, parallel and global search method, which can automatically acquire and accumulate knowledge about search space in the process of search, and control the search process adaptively to get the best solution^33^. This paper adopts GA in curve fitting, and its process is shown in Fig. 18.

**Figure 19:**
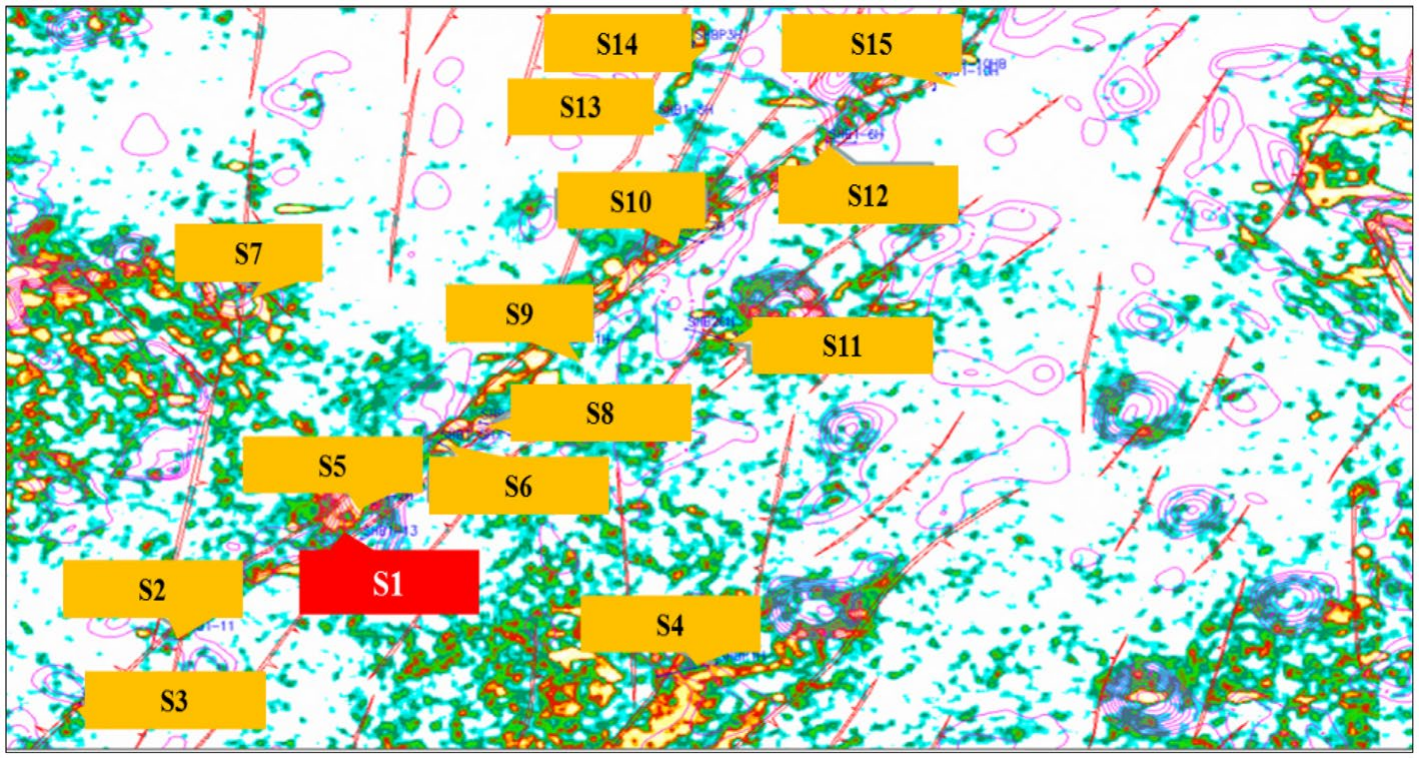
The location of the well S1.

Well S1 is located in the southern part of the Shunbei field, as shown in Fig. 19. The well was drilled on November 12, 2016, and completed on January 6, 2017, with a depth of 5703 m. The length of horizontal section is 639.2 m, with a closed azimuth of 301.4°.

**Figure 20:**
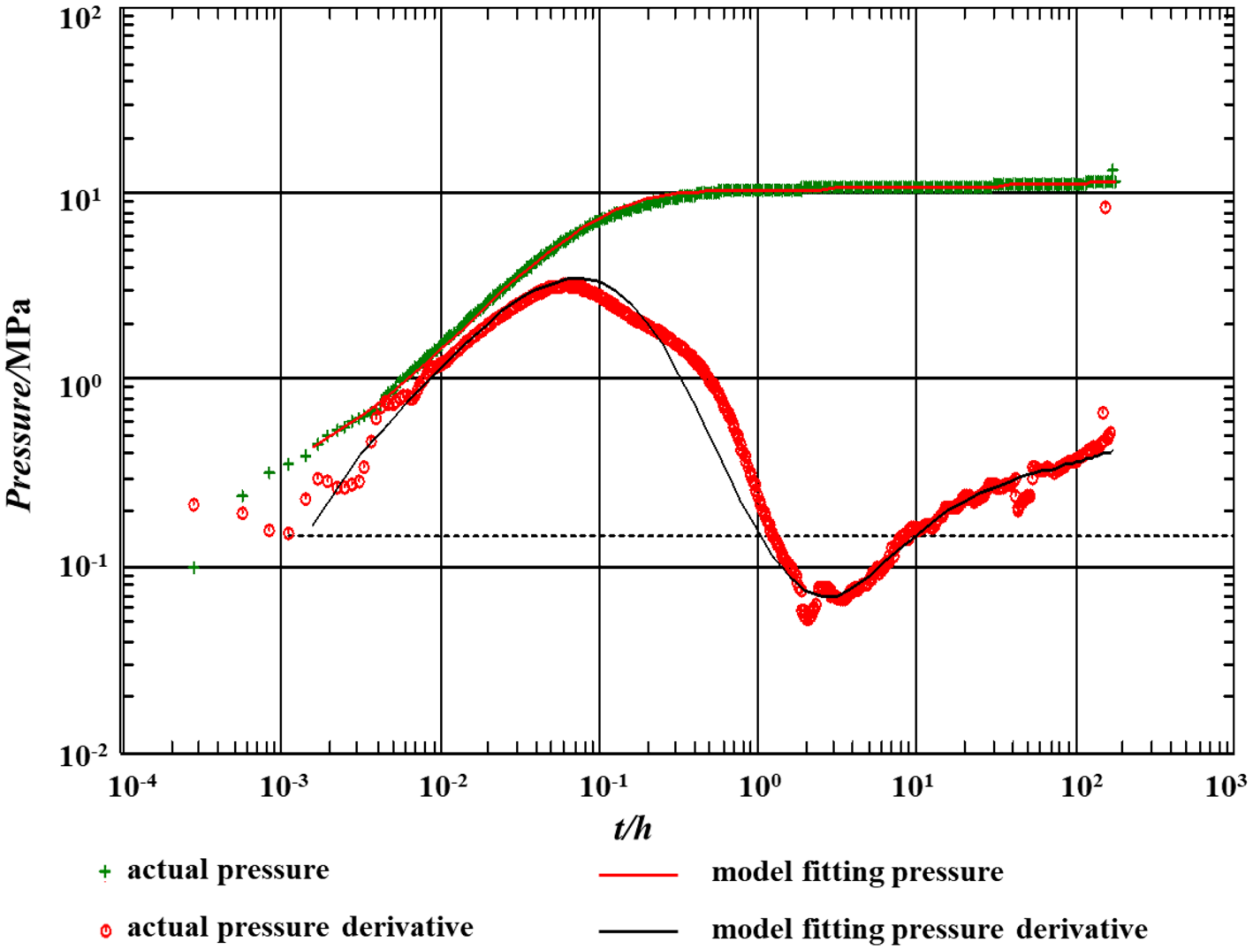
Well test model fitting.

After the well test, well S1 was shut in and pressure recovered was measured on January 17, 2017. The bottom hole pressure was 61.83 MPa/5703 m when shut in and 73.15 MPa after shut in 110 h. The well test model fitting is shown in Fig. 20. Figure 20 shows that the wellbore storage coefficient of well S1 has changed from small to large, resulting in poor fitting of the pressure derivative curve at the beginning. At the same time, the pressure curve does not coincide with the pressure derivative curve, and the slope of the curve is less than 1. In the middle and late stages of percolation, a “concave” appeared in the pressure derivative curve of well S1, reflecting the supply of liquid from karst caves to fractures. Although the well test data is incomplete, the actual value is in good agreement with the calculated value of the model in this paper.

This case confirms that the measured value and the theoretical calculation value are in good agreement. If we ignore the change of the wellbore storage coefficient, the fitting in the early stage will also be better. After considering the fracture orientation of the reservoir, the model in this paper is closer to the actual situation of the reservoir.

Please note that: The biggest drawback of the model in this paper is that it cannot completely solve the bottom hole pressure problem of the multi-cavity system. When the horizontal well does not encounter karst caves, the karst caves in the reservoir are connected to the wellbore through fractures. The model in this paper can solve this situation. However, when a horizontal well is drilled into multiple karst caves, because the karst caves directly supply fluid to the horizontal wellbore, instead of first flowing to the fractures and then from the fractures to the wellbore, it will interfere with the bottom hole pressure response characteristics. In this case, the model in this paper needs to be further improved, and a triple-media seepage model needs to be established to solve this problem.

## Summary and conclusions

A model is derived mathematically to investigate the role of hydraulic fracture properties on the transient bottomhole pressure (BHP) behavior of a horizontal well producing from a tight fracture-cavity reservoir. First, a new point source function for triple-medium fractured-vuggy reservoirs is established. Then, being based on the new point source function, the steps for solving the transient bottomhole pressure (BHP) of horizontal wells are given. Through literature comparison and numerical simulation methods, the rationality of the method in this paper is verified. By drawing typical pressure and pressure derivative curves, the seepage laws of horizontal wells in a tight fracture-cavity reservoir are summarized, and the characteristics of each flow stage are analyzed in detail.

The results identify five flow regimes, namely the wellbore storage stage (*p*_*wD*_ ∼ *t*_*D*_ and *dp*_*wD*_/*dlnt*_*D*_* ∼ t*_*D*_), the karst cave fluid flows to the fracture stage, the radial flow stage of karst cave and fracture system, the matrix fluid flows to the fracture stage and the quasi-steady state stage (*p*_*wD*_ ∼ *t*_*D*_ and *dp*_*wD*_/*dlnt*_*D*_* ∼ t*_*D*_).

The presented results reveal that the influences of fracture number, fracture angle, fracture half-length, skin factor, horizontal well segment length, permeability modulus and horizontal well segment spacing play significant roles on transient bottomhole pressure (BHP) behavior of a horizontal well producing from a tight fracture-cavity reservoir.

The number of fractures and fracture direction mainly affect radial flow stage. In contrast, the length of horizontal subsection and skin factor mainly affect the karst cave fluid flows to the fracture stage. The matrix fluid flows to the fracture stage is more obvious when the fracture half-length and the horizontal segment spacing of the horizontal well are small.

The study indicates that special attention should be paid to reforming the formations at both ends of the horizontal well to eliminate pollution near the wellbore When using horizontal wells to implement reservoir production stimulation measures. When designing a fracturing operation, the perforating gun should be made parallel to the horizontal wellbore as much as possible, and the fracture half-length formed should be greater than 50 m. When designing the fracturing of horizontal wells, it is necessary to increase the heel and toe production sections of horizontal wells, expand the drainage area, and accelerate the supply of fractures to horizontal wellbore, to improve the productivity of horizontal wells.

The developed solution in this paper is useful for well test interpretation through generating a new set of type curves and the accuracy of well test interpretation is improved to a certain extent. It can also be used to analyze the seepage law and explain the reservoir parameters, including fracture number, fracture angle, fracture half-length, skin factor, horizontal well segment length, permeability modulus and horizontal well segment spacing. In other words, the results of this study have practical significance, especially for well testing of hydraulic fractured horizontal wells in fractured-vuggy tight reservoirs.

## Appendix 1 Dimensionless transformation

The dimensionless transformation is defined as follows:

22 $$ N = \sqrt{\frac{abh}{L}}  $$

23 $$ t_{D} = \frac{{k_{if} }}{{(\phi \mu c)_{f + m + v} N^{2} }}t\;P_{Dj} = \frac{{2\pi k_{if} N\left( {p_{i} - p_{j} } \right)}}{QB\mu },{\kern 1pt} {\kern 1pt} {\kern 1pt} {\kern 1pt} j = f/m\;k_{if} = \sqrt[3]{{k_{x} k_{y} k_{z} }} $$

24 $$ r_{D} = \sqrt {\left[ {\left( {\sqrt {\frac{k}{{k_{x} }}} \frac{x}{N}} \right)^{2} + \left( {\sqrt {\frac{k}{{k_{y} }}} \frac{y}{N}} \right)^{2} + \left( {\sqrt {\frac{k}{{k_{z} }}} \frac{z}{N}} \right)^{2} } \right]} $$

25 $$ a_{D} = \frac{a}{N}\;b_{D} = \frac{b}{N}\;h_{D} = \frac{h}{N} $$

26 $$ w_{xD} = \frac{{w_{x} }}{N}\;w_{yD} = \frac{{w_{y} }}{N}\;w_{zD} = \frac{{w_{z} }}{N} $$

27 $$ x_{0D} = \frac{{x_{0} }}{r}\;y_{0D} = \frac{{y_{0} }}{r}\;z_{0D} = \frac{{z_{0} }}{r} $$

28 $$ x_{D} = \sqrt {\frac{k}{{k_{x} }}} \frac{x}{N}\;y_{D} = \sqrt {\frac{k}{{k_{x} }}} \frac{y}{N}\;z_{D} = \sqrt {\frac{k}{{k_{x} }}} \frac{z}{N} $$

29 $$ \omega_{j} = \frac{{(\phi c)_{j} }}{{(\phi c)_{f + m + v} }},{\kern 1pt} {\kern 1pt} {\kern 1pt} {\kern 1pt} {\kern 1pt} {\kern 1pt} j = f/m/v $$

30 $$ \lambda_{vf} = \frac{{\sigma_{v} k_{v} N^{2} }}{{k_{if} }}\;\lambda_{mf} = \frac{{\sigma_{m} k_{m} N^{2} }}{{k_{if} }}\;\lambda_{mv} = \frac{{\sigma_{m} k_{m} N^{2} }}{{k_{v} }} $$

Based on the dimensionless transformation above, Eq. (4) can be summarized as:

31 $$ e^{{ - \Delta p_{f} }} \frac{1}{{r_{D}^{2} }}\frac{\partial }{{\partial r_{D} }}(r_{D}^{2} \frac{{\partial \Delta p_{fD} }}{{\partial r_{D} }}) + \frac{4\pi }{{N^{2} }}f(r_{D} ) = \omega_{f} \frac{{\partial \Delta p_{fD} }}{{\partial t_{D} }} + \omega_{m} \frac{{\partial \Delta p_{mD} }}{{\partial t_{D} }} + \omega_{v} \frac{{\partial \Delta p_{vD} }}{{\partial t_{D} }} $$

where *f(r*_*D*_) is

32 $$ f(r_{D} ) = \frac{1}{{8w_{xD} w_{yD} w_{zD} }}\iiint\limits_{{v_{D} }} {\cos \alpha \cos \beta \cos \chi \cdot \delta \left[ {\left( {r_{D} - r_{0D} } \right)} \right]^{3} }dv_{D} $$

33 $$ \left( {x_{0D} - w_{xD} } \right)\left( {y_{0D} - w_{yD} } \right)\left( {z_{0D} - w_{zD} } \right) \le v_{D} \le \left( {x_{0D} + w_{xD} } \right)\left( {y_{0D} + w_{yD} } \right)\left( {z_{0D} + w_{zD} } \right) $$

Matrix system dimensionless equation:

34 $$ - \omega_{m} \frac{{\partial \Delta p_{mD} }}{{\partial t_{D} }} = \lambda_{mf} (\Delta p_{mD} - \Delta p_{fD} ) $$

Karst cave system dimensionless equation:

35 $$ - \omega_{m} \frac{{\partial \Delta p_{vD} }}{{\partial t_{D} }} = \lambda_{vf} (\Delta p_{vD} - \Delta p_{fD} ) $$

The internal boundary condition:

36 $$ \frac{{d\Delta p_{fD} }}{{dt_{D} }} - r_{D} \frac{{\partial \Delta p_{fD} }}{{\partial r_{D} }}\left| {_{{r_{D} = 1}} } \right. = 1 $$

The outer boundary condition:

37 $$ \Delta p_{fD} (\infty ,t_{D} ) = \Delta p_{vD} (\infty ,t_{D} ) = \Delta p_{mD} (\infty ,t_{D} ) = 0 $$

The initial conditions:

38 $$ \Delta p_{fD} (r_{D} ,0) = \Delta p_{vD} (r_{D} ,0) = \Delta p_{mD} (r_{D} ,0) $$

## Appendix 2 Derivation of solution

The Laplace transformation with respect to *t*_*D*_ is defined as follow:

39 $$ \overline{p} { = }\int_{0}^{{{ + }\infty }} {e^{{ - st_{D} }} } pdt_{D} $$

where *s* is the variable of the Laplace transformation.

The Laplace transform of equation (31), (34) and (35) is obtained as follows:

40 $$ e^{{ - \Delta p_{f} }} \frac{1}{{r_{D}^{2} }}\frac{\partial }{{\partial r_{D} }}\left(r_{D}^{2} \frac{{\partial \Delta \overline{{p_{f} }} }}{{\partial r_{D} }}\right) + \frac{4\pi }{{sN^{2} }}f(r_{D} ) = s\omega_{f} \Delta \overline{{p_{fD} }} + s\omega_{m} \Delta \overline{{p_{mD} }} + s\omega_{v} \Delta \overline{{p_{vD} }} $$

41 $$ - s\omega_{m} \Delta \overline{{p_{mD} }} = \lambda_{mf} (\Delta \overline{{p_{mD} }} - \Delta \overline{{p_{fD} }} ) $$

42 $$ - s\omega_{v} \Delta \overline{{p_{vD} }} = \lambda_{vf} (\Delta \overline{{p_{vD} }} - \Delta \overline{{p_{fD} }} ) $$

Substitute Eqs. (41) and (42) into Eq. (40), we can get:

43 $$ e^{{ - \Delta p_{f} }} \frac{1}{{r_{D}^{2} }}\frac{\partial }{{\partial r_{D} }}\left(r_{D}^{2} \frac{{\partial \Delta \overline{{p_{f} }} }}{{\partial r_{D} }}\right) + \frac{4\pi }{{sN^{2} }}f(r_{D} ) = sf(s)\Delta \overline{{p_{f} }} $$

where

44 $$ f(s) = \omega_{f} + \omega_{m} \frac{{\lambda_{mf} }}{{\lambda_{mf} + \omega_{m} s}} + \omega_{v} \frac{{\lambda_{vf} }}{{\lambda_{vf} + \omega_{v} s}} $$

Equation (43) is a strongly nonlinear partial differential equation, and a perturbation transformation is introduced:

45 $$ \vartriangle p_{f} = - \frac{\ln (1 - \alpha \psi )}{\alpha } $$

The derivative of Eq. (45) with respect to *r*_D_ can be obtained as follows:

46 $$ \frac{{d\Delta p_{f} }}{{dr_{D} }} = \frac{1}{1 - \alpha \psi }\frac{d\psi }{{dr_{D} }} $$

The derivative of Eq. (46) with respect to *t*_D_ can be obtained as follows:

47 $$ \frac{{d\Delta p_{f} }}{{dt_{D} }} = \frac{1}{1 - \alpha \psi }\frac{d\psi }{{dt_{D} }} $$

Substitute Eqs. (46) and (47) into Eq. (43), we can get:

48 $$ \frac{1}{{r_{D}^{2} }}\frac{\partial }{{\partial r_{D} }}\left(r_{D}^{2} \frac{{\partial \Delta \overline{\psi } }}{{\partial r_{D} }}\right) + \frac{4\pi }{{sN^{2} }}f(r_{D} ) = - sf(s)\frac{1}{1 - \alpha \psi }\overline{\psi } $$

In the form of power series, the$\psi$ and $1/\left( {1/\alpha \psi } \right)$ can be rewritten as

49 $$ \psi = \psi_{0} + \alpha \psi_{1} + \alpha^{2} \psi_{2} + \alpha^{3} \psi_{3} + ... $$

50 $$ \frac{1}{1 - \alpha \psi } = 1 + \alpha \psi + \alpha^{2} \psi^{2} + \alpha^{3} \psi^{3} + ... $$

Because of the small permeability modulus $\psi$, scholars believe that the 0-order perturbation solution can meet the needs of engineering calculation. Take the 0-order perturbation solution in Eqs. (49) and (50), and substitute it into Eq. (48), we can get:

51 $$ \frac{1}{{r_{D}^{2} }}\frac{d}{{dr_{D} }}\left(r_{D}^{2} \frac{{\partial \overline{\psi }_{0} }}{{\partial r_{D} }}\right) + \frac{4\pi }{{sN^{2} }}f(r_{D} ) - sf(s)\overline{\psi }_{0} = 0 $$

If:

52 $$ g = r_{D} \overline{{\psi_{0} }} $$

Then Eq. (51) can be rewritten as:

53 $$ \frac{{d^{2} g}}{{dr_{D}^{2} }} - sf(s)g = - \frac{{4\pi f(r_{D} )}}{{N^{2} }}\frac{1}{s} $$

The Equation (53) is a non-homogeneous second-order differential equation. The idea of solving it is: Firstly, find the general solution of the corresponding homogeneous second-order differential equation; secondly, find a special solution of the non-homogeneous second-order differential equation; finally the general solution + special solution is the non-homogeneous solutions of second-order differential equation The corresponding homogeneous equation of Eq. (53) is

54 $$ \frac{{d^{2} g}}{{dr_{D}^{2} }} - sf(s)g = 0 $$

The general solution of Eq. (54) is

55 $$ g = A\exp \left( - r_{D} \sqrt {sf(s)} \right) + B\exp (r_{D} \sqrt {sf(s)} ) $$

Substitute Eq. (55) into Eq. (52), we can get

56 $$ \overline{{\psi_{0} }} = A\frac{{\exp ( - r_{D} \sqrt {sf(s)} )}}{{r_{D} }} + B\frac{{\exp (r_{D} \sqrt {sf(s)} )}}{{r_{D} }} $$

Through internal and external boundary conditions, A and B can be solved:

57 $$A = \frac{1}{{4\pi L^{3} }},{\kern 1pt} {\kern 1pt} B = 0$$

In the same way, a special solution of Eq. (53) can be obtained as:

58 $$ \overline{{\omega_{0} }}^{*} = \frac{{4\pi \ln sf(r_{D} )}}{{N^{2} }} - sf(s) $$

Then, the solution of instantaneous point source in the Laplace space of fracture-cavity triple medium reservoir is:

59 $$ \overline{{\omega_{0} }} = \left[\frac{{4\pi \ln sf(r_{D} )}}{{N^{2} }} - sf(s)\right] + \frac{{\overline{q} }}{{\left[(\phi c_{t} )_{f} + (\phi c_{t} )_{m} + (\phi c_{t} )_{v} \right]}}\frac{{\exp \left( - r_{D} \sqrt {sf(s)} \right)}}{{4\pi L^{3} r_{D} }} $$

## List of symbols

*k*_*x*_, *k*_*y*_ and *k*_*z*_: The permeability in x, y and z directions, μm^2^
*μ*: The viscosity of crude oil, Pa s
∆p: Pressure difference, MPa
*t*: Production time, h
$\phi$: The porosity, decimal
*C*: The compressibility coefficient, MPa^−1^
$\alpha ,\beta ,\chi$: The angle between the fracture and the x, y, z axis, degree
*δ*: The dirac delta function
k_xi_: The initial permeability of the natural fracture system in the x direction, μm^2^
p_i_: The initial formation pressure, MPa
α: The permeability modulus, MPa^−1^
*a*, *b*: The length and width of the reservoir, m
*h*: Effective thickness, m
*L*: Horizontal section length of horizontal well, m
*s*: The variable of the Laplace transformation
(*x*_0_, *y*_0_, *z*_0_): Fracture center coordinates
*w*_x_, *w*_y_, *w*_z_: The projection width of the fracture in the x, y, z direction, m

## Subscript

*m*: The matrix system
*f*: Natural fracture system
*v*: Refers to the karst cave system
